# Population genetic structure, migration, and polyploidy origin of a medicinal species *Gynostemma pentaphyllum* (Cucurbitaceae)

**DOI:** 10.1002/ece3.5618

**Published:** 2019-09-12

**Authors:** Xiao Zhang, Hailun Su, Jia Yang, Li Feng, Zhonghu Li, Guifang Zhao

**Affiliations:** ^1^ Key Laboratory of Resource Biology and Biotechnology in Western China (Ministry of Education) College of Life Sciences Northwest University Xi'an China

**Keywords:** conservation strategy, genetic diversity, *Gynostemma pentaphyllum*, migration, polyploidy origin, population structure

## Abstract

*Gynostemma pentaphyllum*, a member of family Cucurbitaceae, is a perennial creeping herb used as a traditional medicinal plant in China. In this study, six polymorphic nSSR and four EST‐SSR primers were used to genotype 1,020 individuals in 72 wild populations of *G. pentaphyllum*. The genetic diversity and population structure were investigated, and ecological niche modeling was performed to reveal the evolution and demographic history of its natural populations. The results show that *G. pentaphyllum* has a low level of genetic diversity and high level of variation among populations because of pervasive asexual propagation, genetic drift, and long‐term habitat isolation. Results of the Mantel test demonstrate that the genetic distance and geographic distance are significantly correlated among *G. pentaphyllum* natural populations. The populations can be divided into two clusters on the basis of genetic structure. Asymmetrical patterns of historical gene flow were observed among the clusters. For the contemporary, almost all the bidirectional gene flow of the related pairs was symmetrical with slight differences. Recent bottlenecks were experienced by 34.72% of the studied populations. The geographic range of *G. pentaphyllum* continues to expand northward and eastward from Hengduan Mountains. The present distribution of *G. pentaphyllum* is a consequence of its complex evolution. Polyploidy in *G. pentaphyllum* is inferred to be polygenetic. Finally, *G. pentaphyllum* is a species in need of protection, so in situ and ex situ measures should be considered in the future.

## Graphical abstract

In the current study, the genetic diversity and population structure were investigated combining with ecological niche modelling to reveal the evolutionary and population demographic history for natural populations of *Gynostemma pentaphyllum*. We assessed the genetic diversity and population structure of *G. pentaphyllum*, explored the migration, and speculated the formation of polyploidies, as well as provided basic information to formulate the corresponding conservation strategy.

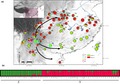

## 1 INTRODUCTION

Population genetic diversity is the product accumulated in the long‐term historical process of evolution in species or populations. It can be used to assess the potential for species survival, adaptation, and development. The evolutionary potential of a species and its ability to mitigate against adverse environmental factors depend not only on the level of genetic variation within the species (genetic polymorphism), but also on the population genetic structure (Li, Liu, Zhao, Su, & Zhao, 2012). Thus, it is necessary to investigate population genetics to evaluate evolutionary processes, and to assess the utilization and conservation of genetic resources. In the past decades, a number of studies of population genetics have used the Himalaya–Hengduan Mountains (HHM) areas and the Qinghai‐Tibetan Plateau (QTP) to examine the effects of orographic uplift and climatic perturbation on plant speciation and population demography (Du, Hou, Wang, Mao, & Hampe, 2017; Liu et al., 2013). In contrast, few studies have been conducted in subtropical China (Sun, Hu, Huang, & Vargas‐Mendoza, 2014; Wang et al., 2015), which consists of the hills and mountains of the Qinling Mountains–Huai River area and the south tropical region of China (Qiu, Fu, & Comes, 2011). Subtropical China is thought to have acted as a refugium for many ancient species during the Pleistocene glacial and interglacial cycles (e.g., Wang, Gao, Kang, Lowe, & Huang, 2009). Many species of this area have unique haplotypes with high levels of genetic diversity. Moreover, the level of genetic differentiation among glacial refugia should be high because of the random fixation of alleles (Hewitt, 2000; Zhang et al., 2015).

*Gynostemma pentaphyllum* is a perennial creeping plant found in subtropical China, Japan, Myanmar, and India (Chen & Gilbert, 2006). In China, it mainly grows near rivers and in the shade of the forests that cover the Yangtze River basin and its southern areas (Chen, 1995). *Gynostemma pentaphyllum* belongs to the Cucurbitaceae family and has 5–7 foliolate leaves. It can reproduce sexually or by clonal growth of rhizomes or bulbils (Gao, Chen, Gu, & Zhao, 1995). Polyploidization is common in *G. pentaphyllum*, which can be diploid, tetraploid, hexaploid, or octoploid (*x* = 11, 2*n* = 22, 44, 66, and 88). However, it is difficult to determine the ploidy based on the morphological features (Gao et al., 1995). At present, it is not known if the polyploid complex of *G. pentaphyllum* is autopolyploid or allopolyploid, and the genetic signature and origin of populations with different ploidies are still unclear. As a traditional Chinese medicinal herb, *G. pentaphyllum* is useful in medical science because it can inhibit the reproduction of tumor cells, regulate lipid metabolism, decrease blood sugar, and enhance immunity (Xie et al., 2010). Thus, most studies of this species have focused on the extraction (Yin, Hu, & Pan, 2004), chemistry, and pharmacology (Razmovski‐Naumovski et al., 2005; Tsai, Lin, & Chen, 2010) of its bioactive components. However, the wild populations of *G. pentaphyllum* have decreased and become fragmented as a consequence of the increased use of natural medicinal herbs and habitat destruction, to the extent that *G. pentaphyllum* has been listed as a Grade II Key Protected Wild Plant Species by the Chinese Government (Yu, 1999). It is therefore imperative to investigate the wild populations of *G. pentaphyllum*, including analysis of their genetic diversity and population structure, to formulate an effective conservation strategy. Existing genetic studies of *G. pentaphyllum* (Jiang, Qian, Guo, Wang, & Zhao, 2009; Pang, Zou, & Xiao, 2006) used RAPD and ISSR molecular markers on relatively small sample sets that did not cover the spatial distribution of *G. pentaphyllum* in subtropical China. The simple sequence repeat (SSR) molecular markers, also known as microsatellites, are codominant molecular markers with putative neutral evolutionary history. They can be used to measure or infer bottlenecks (Spencer, Neigel, & Leberg, 2000), local adaptation (Nielsen, 2005), allelic fixation index (*F*_ST_; Slatkin, 1995), population size (Kohn et al., 1999), and gene flow (Waits, Taberlet, Swenson, Sandegren, & Franzén, 2000).

Furthermore, while paleoecological reconstructions of forest biomes provide fundamental guidance for testable phylogeographic hypotheses, they cannot provide details of population history (Gavin et al., 2014; Qiu et al., 2011). Ecological niche modeling (ENM), which can determine past species distributions, can be used to augment the limited fossil record in East Asia (Wang et al., 2015). Combined with molecular data, ENM can strengthen our understanding of the temporal dimension of population dynamics (Mellick, Lowe, Allen, Hill, & Rossetto, 2012; Scoble & Lowe, 2010).

In the current study, SSR markers were used to investigate the genetic diversity and population structure of *G. pentaphyllum*, and ENM was used to investigate the history of the evolution and demographic structure of natural *G. pentaphyllum* populations in subtropical China. The main objectives of our study were to: (a) assess the level of genetic diversity in natural populations; (b) evaluate the degree of differentiation and structure among populations; (c) explore the origins and migration of *G. pentaphyllum*; (d) speculate on the origin of polyploidy; and (e) provide basic information that can be used to formulate a conservation strategy.

## 2 MATERIALS AND METHODS

### 2.1 Plant sampling

**Figure 1:**
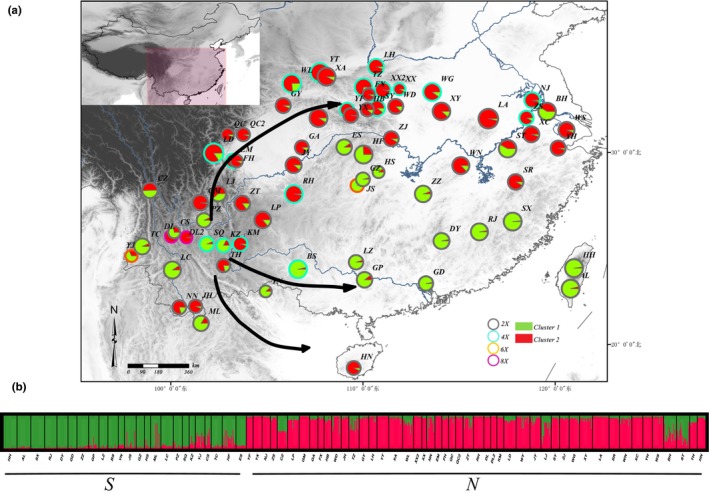
Regional and estimated genetic structure for *K* = 2 for 72 populations of *G. pentaphyllum*. (a) Individual assignment to two clusters for all 72 populations was visualized as pie charts. Each population was partitioned into several colored parts proportionally to its membership in a given cluster; colored rings around the pie charts represented the ploidy of each population (gray: diploid; light blue: tetraploid; orange: hexaploid; purple: octaploid). (b) STRUCTURE plot presented for *K* = 2. Each vertical bar represents a population and its assignment proportion into one of two (colored) population clusters (*K*). The arrows represented the migration paths

Wild *G. pentaphyllum* samples were collected from most of the georeferenced sampling sites; the sample set covers the full longitudinal and latitudinal extent of *G. pentaphyllum* in China (Figure 1; Table A1). Five to twenty‐four individuals were collected randomly from each population, with the number of samples taken dependent on population size. A total of 1,093 individuals in 72 wild populations were collected. Five individuals from each of two *Gomphogyne* populations were selected as outgroups. Fresh leaf materials were dried in silica gel. Root cusp samples were immersed in FAA solution (50 ml of 50% alcohol + 5 ml of glacial acetic acid + 5 ml of 37% formaldehyde) and reserved for further laboratory analysis. A handheld GPS (Garmin eTrex Handheld GPS; Garmin) was used to determine the latitude and longitude of each site. Voucher specimens for the samples were deposited at the Northwest University (Xi'an, Shaanxi).

### 2.2 DNA extraction, amplification, and microsatellite genotyping

Total genomic DNA was extracted using Plant Genomic DNA Kit (TIANGEN Biotech, Beijing Co., Ltd.) following the manufacturer's protocol. Preliminary analyses investigated 14 nSSR and 16 EST‐SSR primers developed in *G. pentaphyllum* (Liao et al., 2011; Zhao, Zhou, Li, & Zhao, 2015), most of them were monomorphic among the populations. At last, six polymorphic nSSR and four EST‐SSR primer pairs (Table 1) were tested to genotype the samples. Polymerase chain reaction (PCR) amplifications were performed using a MyCycler™ Thermal Cycler (Bio‐RAD). A Biometra Thermocycler was used with the following cycling conditions: 94°C for 5 min, 32 cycles of 94°C for 30 s, annealing temperature (Table 1) for 45 s, 72°C for 45 s and an extension step of 72°C for 5 min, and then a final holding temperature of 10°C.

**Table 1:** The primary primer sequences and annealing temperatures of SSR used in the study

Name	ID	Sequence (5′–3′)	Repetitive unit	Repetitive time	Ta (°C)	No. of alleles	PIC
SSR E1	13343	F: TGACCACTCTCAATCTCATCT R: GTTGAACTATGGGAAGAGAGG	TCT	6	51	8	0.712
SSR E2	2782	F: GGTCGAGACTTTTCAGTTTTG R: CATAATCGTTTTGGTGGAAC	GAA	6	52	7	0.655
SSR E3	10765	F: CTCAAACTTATCACCGTCTGA R: ATTCCCCACTCTGTCTCTATC	GAA	5	51	12	0.803
SSR E4	2369	F: GATGGATGAACTAGGCTGTTT R: GCTTCAGGAAATGATCAACA	TGA	5	52	17	0.821
SSR 1	14	F: AAACTTCAGATCTACGCG R: AGAATGGTAGTAGGTTTTG	CT	16	52	13	0.141
SSR 2	16	F: CTGGAATGGATCTTCTTC R: AGCTGTAGTTCGTGGTTA	AG	9	59	15	0.815
SSR 3	17	F: CATAGGCAGCTGTTATTTC R: TGTTGTCAGAAGCATTGG	CT	7	60	10	0.750
SSR 4	21	F: TTCACCACTTATGTCCTA R: GAAAATGAAGGAATTAAG	CT	11	50	17	0.830
SSR 5	25	F: TAAAAGTATGCTACGAGTTCA R: TTATCCCACCATCAGATT	AG	10	53	12	0.196
SSR 6	139	F: AAATTACCAAAGCTACCCTTCT R: TGTAGATCCCAAGCTCCATG	GCT	6	63	8	0.223
Mean						11.9	0.595

The protocols of Sullivan were employed for the nSSR genotyping (Sullivan, 2013). The products of PCR were separated on 12% non‐denaturing polyacrylamide gel (280 V, 50 W, 3 hr) and visualized using 0.1% silver nitrate stained with a PBR322 DNA marker ladder (TIANGEN Biotech, Beijing Co., Ltd.) to assess the length of the DNA bands. Software Quantity One version 4.6.2 (Bio‐Rad Technical Service Department) was used for quantification. Bands were corrected by capillary electrophoresis, based on several individuals for each primer. Capillary electrophoresis was used for the EST‐SSR genotyping. Sample analyses were carried out using the GeneMarker genotyping software (Hulce, Li, Snyder‐Leiby, & Liu, 2011). The raw data were transformed into 1,0 data for further analysis.

### 2.3 Chromosome counts and DNA ploidy‐level estimation

The method of Sang (2002) was used for chromosome counts. However, the chromosomes of this species are very small in size and difficult to identify even under a high‐power microscope. Thus, the PloidyInfer v1.1 (Huang, Ritland, Dunn, & Li, 2019) software was used to confirm and test the ploidy level of every individual of ambiguous genotype in mixed‐ploidy populations. Confounding individuals were removed to make a single ploidy level for each population.

### 2.4 Genetic analysis and population structure

The Micro‐Checker v2.2.3 (Van Oosterhout, Hutchinson, Wills, & Shipley, 2004) software was used to check for large allele and nonamplifying (null) alleles for each microsatellite locus. Hardy–Weinberg equilibrium (HWE) and linkage disequilibrium (LD) were evaluated using FSTAT v2.9.3 (Goudet, 2002). Significance levels were corrected by the sequential Bonferroni method (Rice, 1989), repeated 740 times. The BayeScan v2.1 (Foll, 2012) program was used to detect outlier loci using the data converted by the PGDSpider v 2.0.1.3 (Lischer & Excoffier, 2011) software.

The polymorphism information content (PIC) of each primer was calculated to estimate the allelic variation of SSRs according to the formula:

$$\text{PIC}=1-\sum_{i=0}^{n}Pi^{2}$$

where *Pi* is the frequency of the *i*th allele for a given SSR marker, and *n* is the total number of alleles detected for that SSR marker (Botstein, White, Skolnick, & Davis, 1980). The genetic diversity indices (mean number of alleles; Na, number of effective alleles; Nae, allelic richness; Ar, observed heterozygosity; Ho, expected heterozygosity over all loci; He, gene diversity with unordered alleles; *h*, and individual inbreeding coefficient; *Fi*) of each locus and population were estimated by SPAGeDi (Hardy & Vekemans, 2002), and GenALEx 6.5 (Peakall & Smouse, 2006) was used to estimate Shannon's Information Index (*I*), the percentage of polymorphic loci (PPL), and geographic distance (GGD) among population pairs. The IBM SPSS Statistics v21.0 (SPSS Inc.) software was used to calculate the bivariate correlation between the longitude and latitude and three diversity indices: expected heterozygosity, Shannon's Information Index, and the frequency of private alleles. The Pearson two‐tailed test was used to test correlations, with a significance value of 0.05. The correlation between ploidy and genetic diversity was calculated. The distribution of private allele frequency (Fp) and expected heterozygosity (He) of populations were mapped using the ArcGIS (Esri) program, employing a kriging spherical interpolation method.

The program STRUCTURE v2.3.3 (Pritchard, Stephens, & Donnelly, 2000), which employs Bayesian clustering analysis, was used to analyze the genetic structure, analysis followed the admixture model with independent allele frequencies. Ten independent simulations were run for K from 1 to 12 with 100,000 burn‐in steps followed by 1,000,000 MCMC steps. Two alternative methods were utilized to estimate the most likely number (*K*) of genetic clusters with the online program STRUCTURE HARVESTER (Earl, 2012) by tracing the change in the average of log‐likelihood *L*(*K*) as suggested by Pritchard et al. (2000) and by calculating delta *K* (Δ*K*) according to Evanno, Regnaut, and Goudet (2005). The ArcMap v10.0 and DISTRUCT v1.1 (Rosenberg, 2004) software packages were used to create the distribution of pie charts and bar charts for the data derived from the STRUCTURE analysis.

Analysis of molecular variance (AMOVA) and the fixation indices calculation in Arlequin 3.5 (Excoffier & Lischer, 2010) were used to investigate the extent of genetic differentiation among populations. Calculations were made using four levels of data grouping: (1) species level; (2) ploidy level; (3) two clusters; and (4) five clusters, based on the results of the STRUCTURE analysis, respectively. The significance of the fixation indices was tested using 10^4^ permutations. StAMPP (Pembleton, Cogan, & Forster, 2013), which is an R package for calculation of genetic differentiation and structure of populations with mixed‐ploidy level, was used to calculate the genetic distance and pairwise *F*_ST_.

The online software Isolation by Distance Web Service version 3.23 (http://ibdws.sdsu.edu; Bohonak, 2002; Jensen, Bohonak, & Kelley, 2005) was used to perform a Mantel test (Mantel, 1967) with 10,000 permutations to detect the relationship between genetic distance and geographic distance among populations, and to determine the possible role of isolation by distance (IBD) in the formation of the current population structure. Principal coordinate analysis (PCoA) was performed based on the genetic distance between pairwise populations.

### 2.5 Bottlenecks and formation pattern of population structure

The BOTTLENECK v1.2.02 (Piry, Luikart, & Cornuet, 1999) software was used to detect genetic bottlenecks within all populations and to determine whether populations exhibited a significant number of loci with heterozygosity excess. A “Wilcoxon signed‐rank test” with a two‐phase model of mutation (TPM; Di Rienzo et al., 1994) with 70% stepwise mutations and 30% multistep mutations was used to analyze heterozygosity excess or deficiency. A descriptor of the allele frequency distribution named “mode‐shift indicator” was also used; this method can discriminate between bottlenecked and stable populations (Luikart, Allendorf, Cornuet, & Sherwin, 1998). Ten thousand iterations were performed for each mutational model.

The program 2MOD v0.2 (Ciofi, Beaumontf, Swingland, & Bruford, 1999) was used to estimate the relative likelihoods of immigration–drift equilibrium and drift since a certain time (i.e., the relative effects of gene flow and genetic drift in the current population structure). The program used the settings of Feng et al. (2016), each model was run three times to check whether the MCMC had converged, 100,000 iterations were performed, and the first 10% of iterations in the output were excluded to avoid dependence on initial starting values.

### 2.6 Effective population size and migration

The software Migrate‐n v3.6 (Beerli, 2005) was used to estimate the historical gene flow. The outputs of this software, which calculates the maximum likelihood using the Brownian method and a constant mutation rate (*μ*), include the effective migration rate (*M* = *m*/*μ*, where *m* is the migration rate per generation and *μ* is the mutation rate) paired in both directions, and the theta value (Θ = 4*N*_e_ *μ* where *N*_e_ is the effective population size). Uniform priors and metropolis sampling with 10 short and 1 long chain with 50,000 and 500,000 iterations, respectively, were used to investigate genealogies. Genealogies were sampled 100 steps apart, and the first 1,000 were discarded. The gene flow and number of migrants per population (*N*_m_) were estimated from the values of M and Θ. Before running the program, the results of STRUCTURE were used to define 2 and 5 clusters. The effective population size (*N*_e_) per population was estimated using an average mutation rate for microsatellites of 5 × 10^−4^ (Schlötterer, 2000; Selkoe & Toonen, 2006). The Bayesian‐based program BAYESASS v3.0 (Wilson & Rannala, 2003) was used to estimate contemporary migration rates among the clusters (over the last few generations, mc), with a sampling frequency of 1,000.

### 2.7 Ecological niche modeling

Ecological niche (ENM) modeling was used to predict suitable paleo‐ and current distribution ranges of *G. pentaphyllum* using the Maxent v.3.3.3k (Phillips, Anderson, & Schapire, 2006; Phillips & Dudík, 2008) software. Model inputs included the present geographic distribution and current environmental factors, which were projected back to the Last Glacial Maximum (LGM). The geographic distribution of species was based on the 72 sample sites in this study and 320 records of the species retrieved from the Chinese Virtual Herbarium website (http://www.cvh.org.cn/cms/). Nineteen bioclimatic variables were taken from the WorldClim website (http://www.worldclim.org/; Hijmans, Cameron, Parra, Jones, & Jarvis, 2005). The LGM data used in this study are from the Community Climate System Model (CCSM; Collins et al., 2006). Pairwise correlations were calculated between the 19 variables. Model goodness of fit was evaluated using the area under the receiver operating characteristics curve (AUC). An AUC score above 0.7 was considered to indicate good model performance (Fielding & Bell, 1997).

## 3 RESULTS

### 3.1 Samples and loci assessment

The ploidy of every individual was tested, and mixed ploidies were recognized in a few populations. Confounding individuals were removed to give a single ploidy for each population. Further analyses were performed on the remaining 1,020 individuals from 72 populations.

After Bonferroni corrections, significant deviation from HWE induced by homozygote excess was detected in most populations (Table A2), and the excess was mainly distributed in loci SSRE3, SSR2, and SSRE2. There was no evidence for LD, but 27 null alleles were found to exist in all loci. The null alleles were regarded as missing data in subsequent analysis. The PIC value of 10 loci ranged from 0.141 to 0.830, with an average value of 0.595 (Table 1). Among these, the values of three loci (SSR1, SSR5, and SSR6) were less than 0.5, indicating that the other seven primers are suitable for identification purposes. The mean values of Ar, He, Ho, *F*_ST_, and *G*_ST_ were 3.543, 0.595, 0.334, 0.491, and 0.508, respectively (Table 2, Table A2). These loci have a high level of genetic diversity and differentiation.

**Table 2:** Summary of *F*‐statistics for each locus

Locus	*F*_it_	*F*_is_	*F*_ST_	*G*_ST_
Loc E1	0.404	−0.090	0.453	0.473
Loc E2	0.426	−0.182	0.514	0.538
Loc E3	0.320	−0.159	0.413	0.434
Loc E4	0.486	−0.087	0.527	0.537
Loc 1	0.196	0.134	0.071	0.083
Loc 2	0.439	−0.074	0.477	0.510
Loc 3	0.500	−0.110	0.550	0.519
Loc 4	0.623	0.057	0.600	0.633
Loc 5	0.263	−0.267	0.418	0.480
Loc 6	0.391	−0.035	0.412	0.448
Mean	0.444	−0.092	0.491	0.508
*SE*	0.036	0.027	0.025	0.025

Note

*G*_ST_: equivalent to *F*_ST_ but estimator with different statistical properties.

### 3.2 Genetic diversity of *G. pentaphyllum* populations

**Figure 2:**
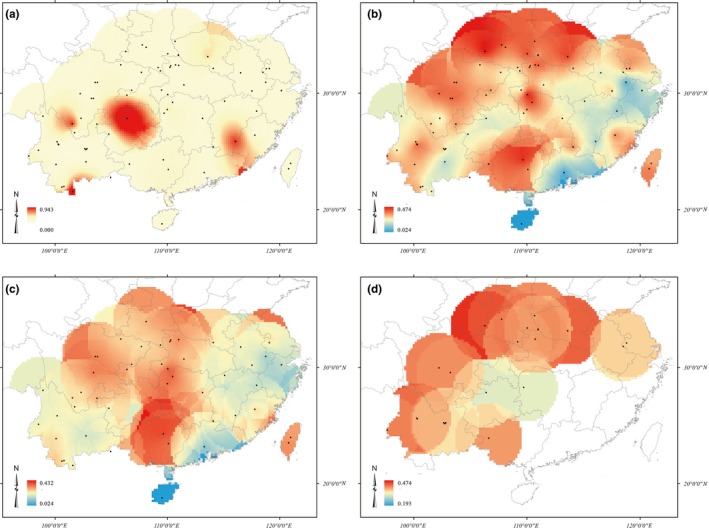
Distribution of *G. pentaphyllum* population diversity based on frequency of private allele and expected heterozygosity. (a) Frequency of private allele (Fp) for all populations, (b) expected heterozygosity (He) for all populations, (c) expected heterozygosity for diploid populations, (d) expected heterozygosity for polyploidy populations. Red represents the higher level and blue represents the lower. Black dots indicate the sampling sites

**Figure 3:**
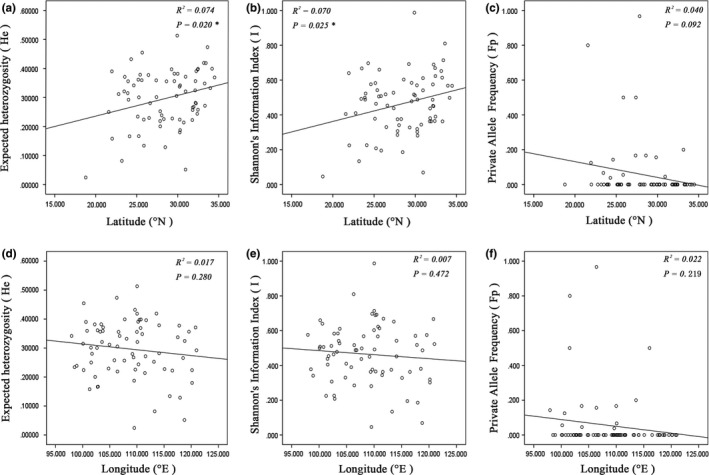
Scatter diagram correlation between the longitude and latitude and three diversity indices, that is, expected heterozygosity, Shannon's Information Index, and private allele frequency, respectively

The level of genetic diversity level in the 72 *G. pentaphyllum* populations was relatively low. The value of He ranged from 0.024 to 0.513, with an average value of 0.297, while Ho ranged from 0.100 to 0.710, with an average value of 0.329. The observed gene diversity is significantly higher than the expected equilibrium gene diversity (*r* = .698, *p* < .01). The value of Ar, *I*, and PPL for each population ranged from 1.09 to 2.74, 0.046 to 0.987, and 10% to 100%, respectively (Table A3). The trends for each genetic parameter were consistent for the 72 populations, of which the HN and HF populations had the lowest and highest genetic diversity, respectively. Private alleles were found in fourteen populations; among them, populations RH and ZT had two private alleles, and the others had one. The 72 populations were divided into 4 groups based on ploidies, and their genetic diversity indices were compared. The genetic diversity of the polyploid populations is greater than that of the diploid populations; the ranking of diversity is octoploid > tetraploid > hexaploid > diploid. The geographic distribution of population diversity based on Fp and He is shown in Figure 2. It is likely that the Qinling–Daba Mountain areas and southwest China are the center of genetic diversity for *G. pentaphyllum*. The correlations between genetic diversity indices and ploidy were calculated, and only Nae and He showed a significant positive correlation (Table 3). Correlations were calculated between three diversity indices (He, I, and Fp) and the longitude and latitude. The only positive correlations of significance were between the He and *I* parameters and latitude (Figure 3).

**Table 3:** The correlation between genetic diversity indices and ploidy of each population

	Fp	Nae	AR	He	*I*	PPL
Ploidy						
Pearson's correlation coefficient	−0.009	0.321	0.191	0.291	0.189	0.161
*p*‐Value	.938	.006*	.108	.013*	.112	.177

* *p* < .05.

### 3.3 Genetic structure and divergence

**Figure 4:**
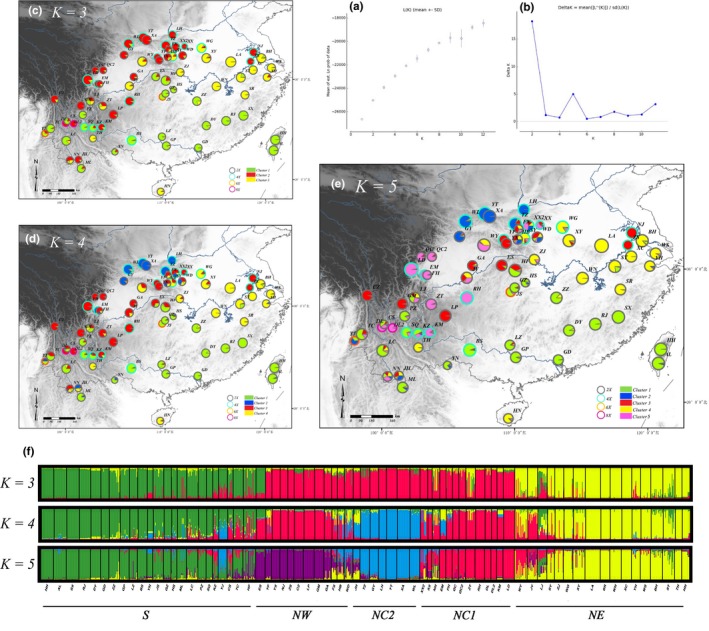
Genetic structure for *K* = 3 to *K* = 5 for 72 populations of *G. pentaphyllum*. Bayesian inference analysis for determining the most likely number of clusters (*K*) for the distribution of (a) the likelihood L(*K*) values and (b) Δ*K* values was presented for *K* = 1–12 (10 replicates per *K*‐value). (c–e) Individual assignment to 3–5 clusters for all 72 populations was visualized as pie charts. Each population was partitioned into several colored parts proportionally to its membership in a given cluster; colored rings around the pie charts represented the ploidy of each population (gray: diploid; light blue: tetraploid; orange: hexaploid; purple: octaploid). (f) STRUCTURE plots were presented for *K* = 3 to *K* = 5, respectively. Each vertical bar represents a population and its assignment proportion into one of three to five (colored) population clusters (*K*)

STRUCTURE analysis clearly differentiated the populations into two clusters: north cluster (N) and south cluster (S) with little admixture (Figure 1). There is a clear peak in the value of DK at *K* = 2 and a small peak at *K* = 5 (Figure 4b). Some populations also form clusters at *K* = 3 or *K* = 4, but these are inconsistent with high variance (Figure 4c,d). The south cluster (S) was stable at higher K values, but the north cluster (N) showed some evidence for partitioning into further clusters: a northwest cluster (NW), a north‐central cluster 1 (NC1), a north‐central cluster 2 (NC2), and a northeast cluster (NE). Most of the ploidy populations fell into clusters with similar genotypes, rather than clusters with any other groups or a single group, except for two tetraploid populations in the northeast cluster (NJ and ZS).

**Figure 5:**
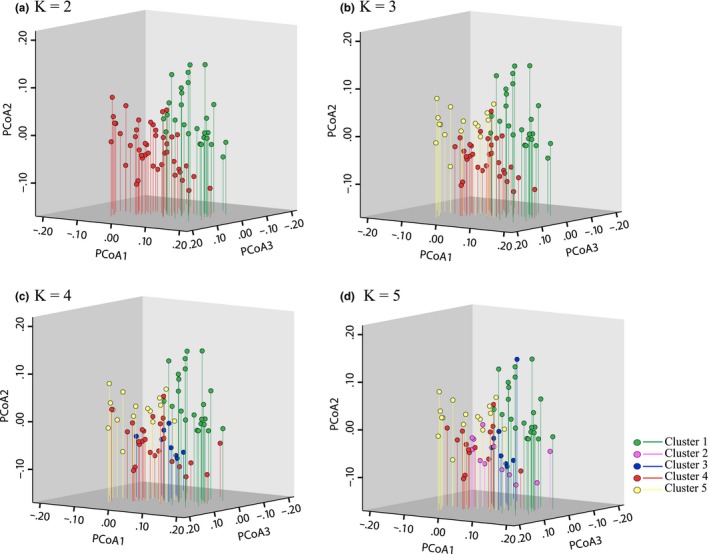
PCoA graph of *G. pentaphyllum*. Principal coordinate analysis of pairwise distances between populations of *G. pentaphyllum*. Percentage of variation explained by the first 3 axes were 13.54%, 11.24%, and 8.60%, respectively

**Figure 6:**
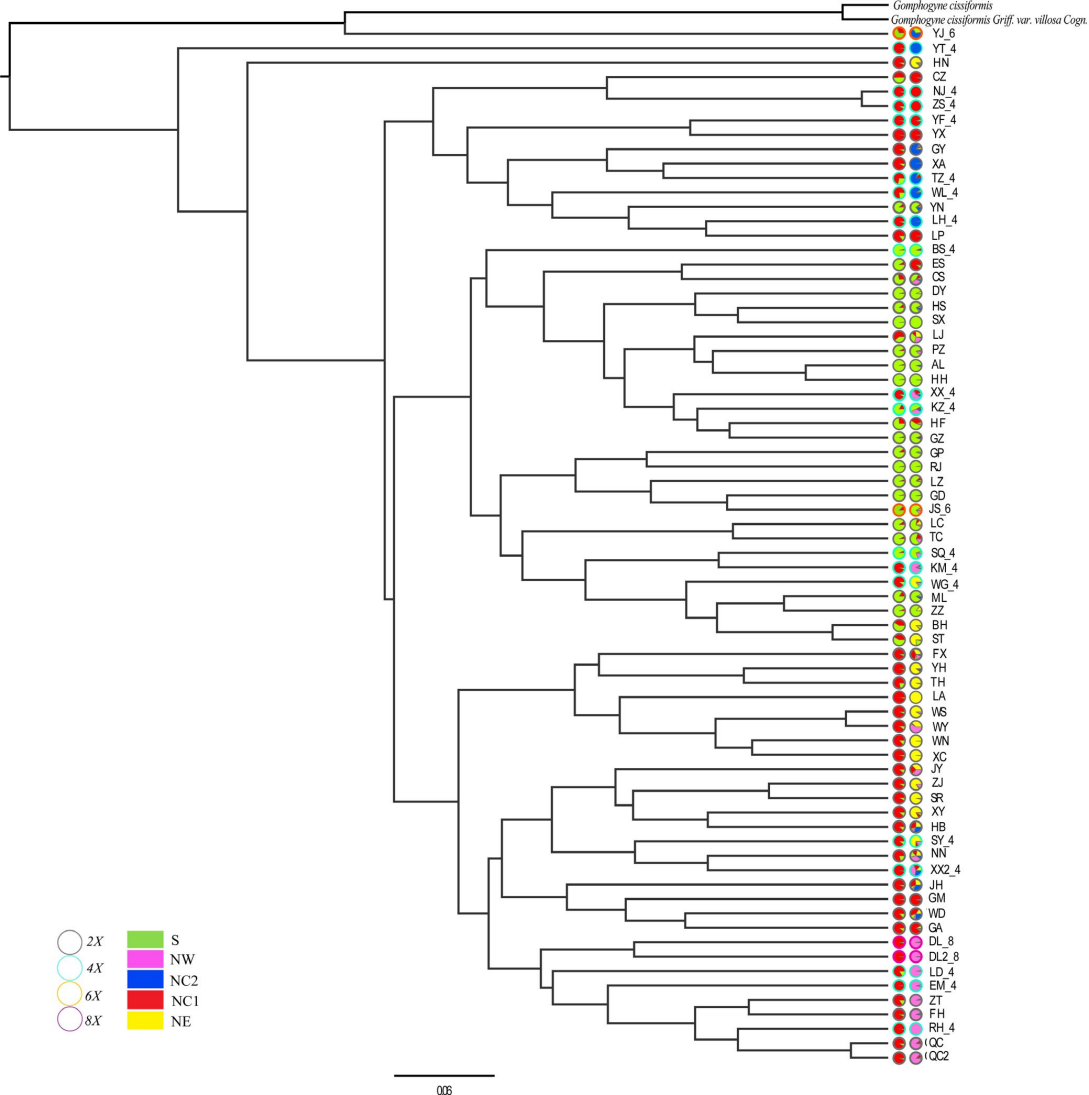
The UPGMA clustering tree of 72 populations of *G. pentaphyllum*. Individual assignment to two to five clusters for all 72 populations was visualized as pie charts. Each population was partitioned into several colored parts proportionally to its membership in a given cluster; colored rings around the pie charts represented the ploidy of each population (gray: diploid; light blue: tetraploid; orange: hexaploid; purple: octaploid)

The relationships between populations based on PCoA plots of pairwise Euclidean distances are consistent with the results of the STRUCTURE analysis. 31.38% is accommodated by the first three components, which separate all populations into their respective groups (Figure 5). Components PC1, PC2, and PC3 account for 13.54%, 11.24%, and 8.60% of the total variance, respectively. The UPGMA tree based on a matrix of *Nei's* genetic distance among the 72 populations divided the accessions into five main branches, with three additional subclusters within those branches (Figure 6). The branches and clusters are consistent with those of the STRUCTURE analysis.

**Figure 7:**
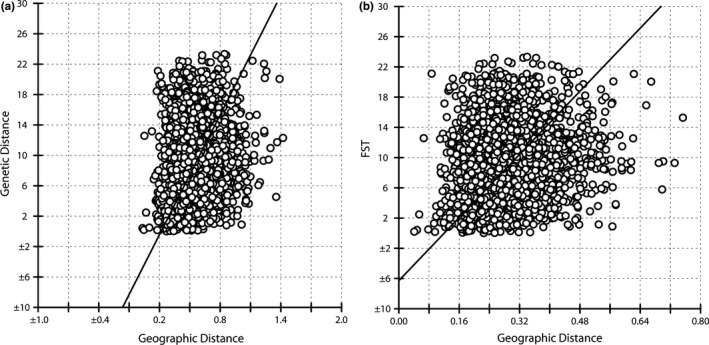
Results of Mantel test. (a) *Nei's* genetic distance versus geographic distance; (b) *F*_ST_ values versus geographic distance

The results of AMOVA reveal that the genetic variation is mostly within populations (Table 4). The percentage of variation within populations at species level, two and five clusters, and ploidy level are 50.42%, 48.19%, 49.22%, and 49.71%, respectively. The corresponding *F*_ST_ values are 0.496, 0.518, 0.508, and 0.503. Results of the Mantel test on the 72 populations (Figure 7) show that there is a significant linear relationship between Nei's genetic distance and geographic distance (*r* = .1518, *p* < .001) and *F*_ST_ value and geographic distance (*r* = .1564, *p* < .001). These results indicate that the genetic diversity and variation are related to geographic distribution.

**Table 4:** Results of AMOVA for the populations of *G. Pentaphyllum*

Source of variation	*df*	Sum of squares	Variance components	Percentage of variation	Fixation indices
(1) Populations					
Among populations	71	3,053.739	1.46669 Va	49.58	*F*_ST_: 0.496
Within populations	1,968	2,935.5	1.49162 Vb	50.42	
Total	2,039	5,989.239	2.95831		
(2) Two clusters					
Among groups	1	305.057	0.26780 Va	8.65	*F*_CT_: 0.087
Among populations within groups	70	2,748.682	1.33611 Vb	43.16	*F*_SC_: 0.473
Within populations	1,968	2,935.5	1.49162 Vc	48.19	*F*_ST_: 0.518
Total	2,039	5,989.239	3.09553		
(3) Five clusters					
Among groups	4	677.497	0.33604 Va	11.09	*F*_CT_: 0.111
Among populations within groups	67	2,376.242	1.20302 Vb	39.69	*F*_SC_: 0.446
Within populations	1,968	2,935.5	1.49162 Vc	49.22	*F*_ST_: 0.508
Total	2,039	5,989.239	3.03068		
(4) Four ploidies					
Among groups	3	179.266	0.07368 Va	2.46	*F*_CT_: 0.025
Among populations within groups	68	2,874.473	1.43553 Vb	47.84	*F*_SC_: 0.490
Within populations	1,968	2,935.5	1.49162 Vc	49.71	*F*_ST_: 0.503
Total	2,039	5,989.239	3.00083		

Note

*F*_ST_ = *F*_SC_ + *F*_CT_.* F*_ST_ = *F*_SC_ + *F*_CT_.

Abbreviations: *df*, degrees of freedom; PV, percentage of variation; SS, sum of squares; VC, variance components; Φ_CT_, differentiation among groups within three species; Φ_SC_, differentiation among populations within species; Φ_ST_, differentiation among populations within three species.

### 3.4 Effective population size and population history

Our study indicated an asymmetrical pattern of historical gene flow among clusters. When the 72 populations are divided into two clusters, the mean migration rate (M) from the north (N) to the south (S) clusters is 11.323 and from S to N is 92.507. The gene flow (*N*_m_) between the two clusters is also asymmetrical (Table 5). The value of *M* between pairs of clusters varies from 9.104 to 36.299 migrants when the populations are divided into five clusters. The effective population size ranges from 245 individuals in the south cluster (S) to 415 individuals in the northeast cluster (NE; Table 6). The highest value of *N*_m_ was 4.680, calculated for migration from the south (S) to north‐central 1 (NC1) cluster. Bidirectional contemporary gene flows of the related pairs were symmetrical, with slight differences. The highest migration rate (0.157) was calculated for migration from the NW cluster to the S cluster; the S cluster provided less immigrations.

**Table 5:** Estimates of migration rate (*M*) among two and five clusters

**Two clusters**					
Migrate‐n	N	S	BAYESASS	N	S
N	(–)	**92.507 (91.319**–**93.702)**	N	(–)	0.232
S	11.323 (11.011–11.639)	(–)	S	0.114	(–)
**Five clusters**					
	S	NC1	NC2	NW	NE
Migrate‐n					
S	(–)	**36.299 (35.614–36.989)**	14.163 (13.779–14.551)	9.2169 (8.920–9.518)	10.263 (9.977–10.551)
NC1	11.929 (11.601–12.260)	(–)	12.402 (12.048–12.761)	12.070 (11.731–12.412)	12.021 (11.713–12.332)
NC2	8.885 (8.601–9.173)	9.104 (8.765–9.448)	(–)	13.3614 (13.00–13.726)	13.277 (12.950–13.608)
NW	9.491 (9.196–9.790)	13.804 (13.390–14.226)	10.160 (9.836–10.489)	(–)	12.122 (11.814–12.433)
NE	8.437 (8.160–8.716)	10.765 (10.397–11.137)	7.7411 (7.453–8.033)	7.652 (7.385–7.922)	(–)
BAYESASS					
S	(–)	0.041	0.042	0.042	0.065
NC1	0.002	(–)	0.041	0.043	0.053
NC2	0.002	0.042	(–)	0.042	0.075
NW	**0.157**	0.041	0.042	(–)	0.009
NE	0.002	0.042	0.042	0.040	(–)

Note

Asymmetrical gene flow was shown in bold. Values in parentheses brackets represented the 5% to 95% confidence intervals (CI). Directionality of gene flow was read among clusters on the left being the source populations, whereas geographic units on top were the recipient populations.

**Table 6:** The effective size of population per cluster and gene flow among all clusters

	Θ	*N*_e_	*M*	*m*	*N*_m_
Two clusters					
S → N	0.497	248.640	11.323	0.0057	1.408
N → S	0.482	241.210	92.507	0.0463	**11.157**
Five clusters					
NC1 → S	0.491	245.370	11.929	0.0060	1.463
NC2 → S			8.885	0.0044	1.090
NW → S			9.491	0.0047	1.164
NE → S			8.437	0.0042	1.035
S → NC1	0.516	257.840	36.299	0.0181	**4.680**
NC2 → NC1			9.104	0.0046	1.174
NW → NC1			13.804	0.0069	1.780
NE → NC1			10.765	0.0054	1.388
S → NC2	0.518	258.970	14.163	0.0071	1.834
NC1 → NC2			12.402	0.0062	1.606
NW → NC2			10.160	0.0051	1.316
NE → NC2			7.741	0.0039	1.002
S → NW	0.584	292.240	9.217	0.0046	1.347
NC1 → NW			12.070	0.0060	1.764
NC2 → NW			13.361	0.0067	1.952
NE → NW			7.652	0.0038	1.118
S → NE	0.830	415.140	10.263	0.0051	2.130
NC1 → NE			12.021	0.0060	2.495
NC2 → NE			13.277	0.0066	2.756
NW → NE			12.122	0.0061	2.516

Note

*M* was mean effective migration rate (*M* = *m*/*μ*); *N*_e_ was the effective size of population; *m* was the migration rate per generation; Θ = 4*N*_e_ *μ*; *N*_m_ was the gene flow or number of migrants per population, and here, *μ* was the mutation rate using the value 5 × 10^−4^. Arrows showed the direction from one cluster to the other.

**Figure 8:**
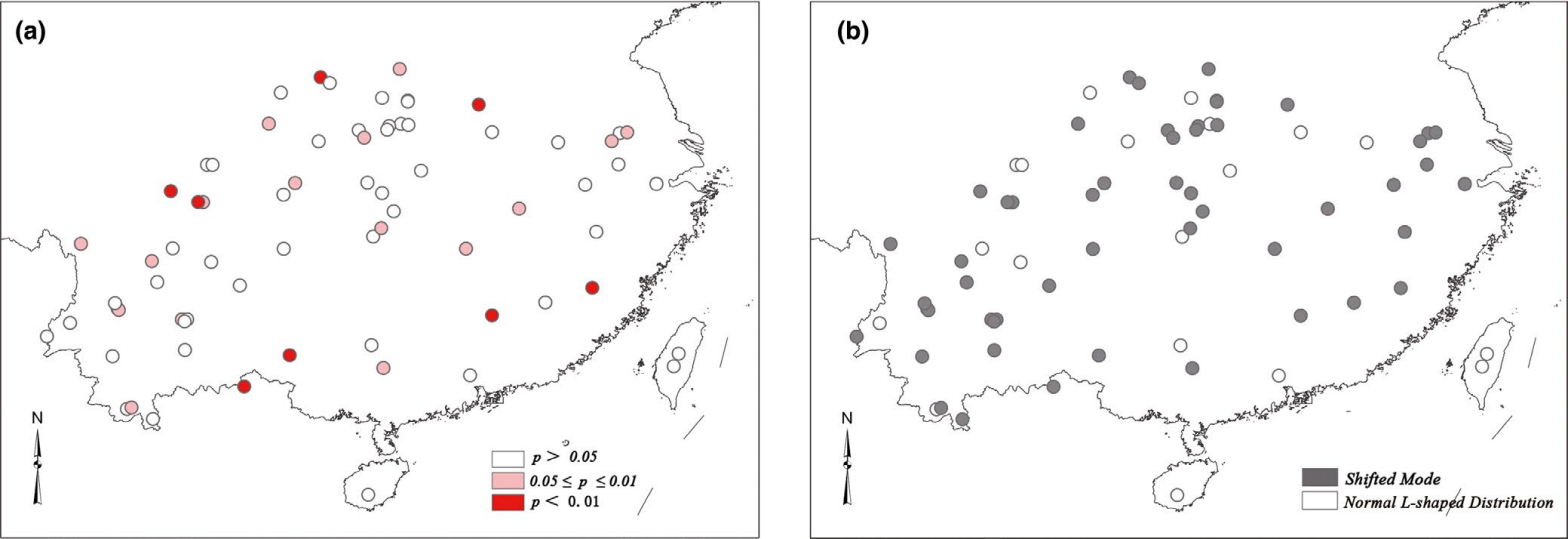
The distribution of bottlenecked populations with four different methods in the TPM model. (a) Wilcoxon test; (b) mode‐shift indicator. Color scales refer to significant level of each population experienced recent bottleneck and results of the mode‐shift indicator

Results of the genetic bottleneck analysis indicate that 34.72% of the populations (25 out of 72) have a high probability of genetic bottleneck (*p* < .05) and that 52 populations show a shifted mode in mode‐shift indicator (Table A3; Figure 8). These populations are inferred to have experienced recent bottlenecks. Results of the 2MOD analysis suggest that a drift model rather than gene flow–drift led to the current population structure (*p* = 1.0, Bayesian factor = 100,000).

### 3.5 Ecological niche modeling

Six bioclimatic variables were selected out of 19 for the ENM (Table 7). The highest contribution rate was by precipitation of warmest quarter (Bio18) at 52.4%, and the most important was temperature seasonality (Bio4), with an important coefficient of 33.9%. Analysis of correlations between the six variables shows no significant correlation, so these variables could be used for further analyses.

**Table 7:** Information of the six ecological variables

Name	Contribution rate (%)	Significance index	Ecological variables
Bio2	2.0	5.0	Mean monthly temperature range
Bio4	7.6	33.9	Temperature seasonality (STD*100)
Bio9	6.7	4.2	Mean temperature of driest quarter
Bio14	1.0	4.6	Precipitation of driest month
Bio15	6.7	12.9	Precipitation seasonality (CV)
Bio18	52.4	18.8	Precipitation of warmest quarter

**Figure 9:**
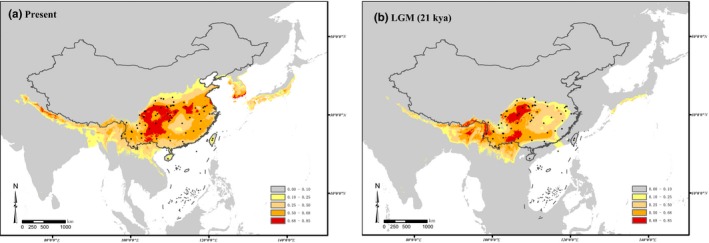
Species distribution modeling using maximum entropy modeling of *G. pentaphyllum*. Predicted distributions were shown for two periods, that is, (a) the present time and (b) the LGM (21,000 years before present) periods. Color scales refer to logistic probability of occurrence, and black dots indicate the sampling sites

The AUC value, based on 10 times repeat, was 0.987, with a standard deviation of 0.003. The calculated distribution under the current climatic conditions is generally similar to the known distribution (Figure 9a), while the predicted suitable habitats for *G. pentaphyllum* in the LGM periods are limited to the Himalayas and Qinling Mountains in southwest and central China (Figure 9b). It is inferred that *G. pentaphyllum* has expanded continuously since the LGM, with an increase in the geographic range to the north and east, including expansion onto the Korean Peninsula and south Japan Islands.

## 4 DISCUSSION

### 4.1 Genetic divergence and diversity

Genetic diversity of a species reflects its evolutionary potential and allows for evolution and adaptation. The more abundant the genetic variation of a species is, the more adaptable it is. Thus, it is necessary to study the genetic diversity of a species to understand its biological properties (Grant, 1985). Subtropical China was a Pleistocene refugium for many ancient species during the Pleistocene glacial and interglacial cycles (e.g., Wang et al., 2009). Species in this region commonly have unique haplotypes, and the level of genetic differentiation among glacial refugia is usually high because of random allele fixation (Hewitt, 2000; Zhang et al., 2015).

In the current study, six SSR and four EST‐SSR markers were used to evaluate the population genetics of a large number of *G. pentaphyllum* populations across its distribution range in subtropical China. The average level of genetic diversity of *G. pentaphyllum* is relatively low (He = 0.297, Ho = 0.329, *I* = 0.465, and PPL = 71.81%, Table A3), though the observed level of genetic diversity is significantly higher than the expected value (*r* = .698, *p* < .01). The trend of these indexes is fairly similar; the lowest and highest diversity were found in the HN and HF populations, respectively. In contrast, Wang, Zhang, Qian, and Zhao (2008) reported that the genetic diversity of 14 *G. pentaphyllum* populations was high (PPL = 96.39%, *I* = 0.407, He = 0.262), based on a study of ISSR markers. The difference may relate to the reproductive attributes of this species, the sample size, and/or the characteristics of the molecular markers. *G. pentaphyllum* is a perennial dioecious herbaceous plant that can be pollinated by insects or propagate asexually by rhizomes or bulbils. In the long term, asexual propagation would lead to a reduction of genetic differences among individuals within populations and enhance differences between populations. Moreover, insects' activity can increase the gene flow among individuals. The results of the AMOVA suggested that the maximum contribution rate is within populations. However, there was also a greater degree of variability among populations (Table 4). Previous studies have suggested that small populations commonly experience serious genetic drift and long‐term habitat isolation might intensify this effect, leading to genetic differentiation among populations by reducing the level of genetic diversity within population (Ellstrand & Elam, 1993). A species with low genetic diversity lacks the evolutionary flexibility to cope with a changing ecological environment and is passive in longer‐term evolutionary processes (Genton, Shykoff, & Giraud, 2005). Thus, a species with low level of genetic diversity is relatively more vulnerable to get extinction (Chen et al., 2015; Knox, Bezold, Cabe, Williams, & Simurda, 2016; Nolan, Noyes, Bennett, Hunter, & Hunter, 2010). In contrast, a high level of genetic diversity tends to be associated with successful ecological adaptation (Ortego, Noguerales, Gugger, & Sork, 2015).

### 4.2 Genetic structure of *G. pentaphyllum*

Genetic distance is commonly used to describe the genetic structure of a population and the differences among populations (Nei, 1972). Among the 72 populations in this study, the highest genetic distance was between the GD and YT populations (genetic distance = 1.42) which are almost the most extreme southernmost and northernmost populations on the mainland. Results of the Mantel test shown that the genetic distance and geographic distance are significantly correlated (*r* = .1518, *p* < .001). It is speculated that the differentiation of populations might be related to the species' asexual reproductive characteristics, geographic isolation, and human activity. *G. pentaphyllum* has been overexploited in recent years, so the natural resources are becoming scarce. Moreover, many of the populations have a fragmented distribution, which also contributes to the genetic differentiation of *G. pentaphyllum*. Habitat fragmentation, therefore, has consequences for the genetic structure of species as well as for the ecological processes, abiotic factors, and the quantity and structure of species that make up an ecosystem (Saunders, Hobbs, & Margules, 1991; Templeton, Shaw, Routman, & Davis, 1990; Young, Boyle, & Brown, 1996).

Patterns in genetic structure are produced by evolutionary and demographic processes at different temporal scales (Morris, Ickert‐Bond, Brunson, Soltis, & Soltis, 2008). Factors such as mutation, migration, natural selection, and genetic drift, as well as the evolutionary history and biological characteristics of the species, combine to produce a nonrandomly distributed pattern of genetic variation in space and time. The evolutionary potential of a species or populations depends to a large extent on the genetic structure of the population (Loveless & Hamrick, 1984). The results of the STRUCTURE analysis performed for this study indicate that the most likely genetic structure of the 72 studied populations is either two or five clusters (Figures 1 and 4). With two clusters (*K* = 2), the populations fall into two groups located in the north and south of the study area, with mixed in the west. Based on the assumption that southwest China is the origin and diversity center of *Gynostemma* species (Chen, 1995), we speculate that the cradle of diversity for *G. pentaphyllum* was in southwest China. With five clusters (*K* = 5), the five clusters are not geographically independent, and there is mixing in some areas, for example, the Hengduan Mountains and Qinling–Daba Mountains. An exception is provided by the HN population, which is located in the southernmost extreme of the distribution area, but groups with the eastern populations. It is possible that this population originated in southwest China but experienced a similar evolutionary history to the eastern group. Some populations consisting of single genetic component (i.e., SX, DL, and LA population) might have experienced significant bottleneck or founder effects.

### 4.3 Gene flow, migration, and diffusion

Mutation and genetic drift lead to genetic differentiation of local populations, and gene flow might promote evolution by spreading new genes (Slatkin, 1987) and producing changes in the spatial distribution and genetic structure of species. In sessile organisms, such as plants, gene flow occurs mainly through pollen and seed dispersal (Robledo‐Arnuncio, Klein, Muller‐Landau, & Santamaría, 2014; Slatkin, 1985). However, factors related to breeding (i.e., outcrossing and self‐fertilization rates), the mode of reproduction (i.e., biparental inbreeding and clonal propagation), and external factors (i.e., a capricious climate, uplifted mountains, broad rivers, wind direction, and animal activity) can facilitate or hinder the gene flow of plants (Robledo‐Arnuncio et al., 2014).

A previous study has suggested that the effective gene flow for *G. pentaphyllum* (*N*_m_ = 0.0622) is much less than one successful migrant per generation (Wang et al., 2008). In contrast, the results of this study suggest a higher rate of gene flow (*N*_m_ > 1). A possible reason for the discrepancy is that the large number of samples and sampling strategy in this study reduced the geographic distances among the populations. *G. pentaphyllum* is a perennial herbaceous plant that is dioecious and pollinated by insects. Pollen flow mediated by insects can promote gene exchange among adjacent populations and individuals. Moreover, according to the Bayesian clustering results, the clusters were not completely independent, but show mosaic phenomena in some areas where the genetic diversity was also abundant, such as the Hengduan Mountains and Qinling–Daba Mountains. It is, therefore, possible that high genetic diversity can be used as evidence for frequent gene flow.

The southwest region of China is the current center of distribution and diversity center for *G. pentaphyllum* (Chen, 1995), and Wang et al. (2008) suggested that the species originated in the area around the Hengduan Mountains. This suggestion is consistent with our results; populations from the Hengduan Mountains displayed components from all Bayesian clusters. Furthermore, the results of ENM suggest that populations from the Hengduan Mountains area are relatively old. We concur with Wang et al. (2008) that *G. pentaphyllum* originated in the Hengduan Mountains area, and speculate that the species expanded northward and eastward along three trajectories. The first trajectory was along the Hengduan Mountains and the edge of Sichuan Basin to the north, through the Qinling–Daba Mountain area, and then eastward, though the eastward spread was affected by the east–west mountain ranges. The populations of this cluster are mostly distributed through mountain areas with a complex topography and varied climate, so new clusters formed during the migration process. Populations on the plains in the east of China mostly fall into a single cluster. The second trajectory was from the southwest of China toward the east. The populations in this cluster are similar to each other, and their compositions are stable. The landforms of eastern and southern China are mostly plains and low hills; these environments are not conducive to the production of new genotypes, and most of the populations in this region experienced bottlenecks, resulting in the reduction of genetic diversity. The effective size of the populations that experienced bottlenecks is usually small, and the number of alleles and heterozygosity is expected to be correspondingly decreased. However, the observed heterozygosity is greater than the heterozygosity calculated from the number of alleles using the mutation–drift equilibrium; this phenomenon is known as heterozygosity excess (Piry et al., 1999). The third trajectory was from the source to Hainan Island, through northern Vietnam and south China.

Results of the ENM analysis also suggest that *G. pentaphyllum* has expanded its distribution range continuously since the last interglacial period (LIG; Figure 9). Indeed, the genus *Gynostemma* is thought to have originated in “West Sichuan Central Yunnan old land,” while southwest China is its modern center of distribution and diversity (Chen, 1995). Moreover, the recent expansion of *G. pentaphyllum* populations in China is from the southwest to the east and north. The north–south asymmetrical gene flow documented by this study is significantly greater than the flow from south to north. The largest recent gene flow was from the NW cluster to the S cluster; this observation suggests that the southern populations are of recent origin.

### 4.4 Origin of polyploidization

Polyploidization is one of the most important evolutionary characteristics of plant species and a major driving force for the high diversity of angiosperms (Otto & Whitton, 2000; Soltis & Soltis, 1999). Approximately 47% of the angiosperm species and 80% of ferns have undergone polyploidization processes in their evolutionary history (Cui et al., 2006; Soltis, 2005). Compared with the diploid species, polyploid species may have broader niches and/or larger distribution ranges (Ehrendorfer, 1980; Li, Wan, Guo, Abbott, & Rao, 2014; Parisod & Besnard, 2007; Ramsey & Ramsey, 2014; Tremetsberger, König, Samuel, Pinsker, & Stuessy, 2002), exhibit increased vigor and competitiveness (te Beest et al., 2011; Lumaret, Guillerm, Maillet, & Verlaque, 1997; Maceira, Jacquard, & Lumaret, 1993; Schlaepfer, Edwards, & Billeter, 2010), and show a preference for distinct habitats (McIntyre, 2012; Ramsey, 2011). Polyploid plants often originate from diploid ancestors, and their origin is often associated with dramatic climate fluctuations and changes in the geological environment (Parisod, Holderegger, & Brochmann, 2010). Previous studies suggest that polyploidization occurred throughout the Quaternary period, and many plant groups exhibit high degrees of polyploidy (Brysting, Oxelman, Huber, Moulton, & Brochmann, 2007). For example, a study of five fragments of chloroplast DNA sequence from diploid–tetraploid complex of *Allium przewalskianum* in the Qinghai–Tibet Plateau and adjacent areas concluded that the tetraploid population of the species originated from its diploid ancestor at least eight separate times and that it had undergone at least one geographic expansion in the origin of the polyploidy complex (Wu, Cui, Milne, Sun, & Liu, 2010). In general, the derivation of polyploidies from different diploid ancestors induces a high level of genetic variation and population differentiation in the polyploid species, which increases the genetic diversity of polyploidies through hybridization and genomic recombination events from the autopolyploid. The results of this study show that polyploid populations have high levels of genetic diversity.

The genus *Gynostemma* might have originated from the “West Sichuan Central Yunnan old land” in early Tertiary (Chen, 1995). Thus, *G. pentaphyllum* probably experienced the effects of severe climate instability and changes to its geological environment during the Quaternary glacial–interglacial period. Polyploidization in natural populations of *G. pentaphyllum* has occurred throughout its long history, including during periods of migration and diffusion. Most of the polyploid populations in this study occur on the edge of the Sichuan Basin and in the Qinling–Daba Mountain area, where the topography and geological history are complex. It is speculated that these populations were affected by changes to the geology and climate. Moreover, the polyploid populations are commonly fragmented and occur in moist forests; it is proposed environmental changes and migration of the species drove the emergence of polyploidies in *G. pentaphyllum*.

The results of this study suggest that *G. pentaphyllum* is autopolyploid (Jiang et al., 2009). The results of both Bayesian clustering and UPGMA tree showed that most polyploid populations in this study were divided into the same cluster as their geographically adjacent diploid populations. Most of the polyploid populations have components in common with neighboring diploid populations, rather than forming a single cluster. Therefore, the polyploid populations are likely to have originated from the adjacent diploid populations and have coexisted with their diploid parents. The origin of polyploidy in *G. pentaphyllum* is therefore inferred to be polygenesis. Similar result was also found in the study of *Galax urceolata* (Servick, Visger, Gitzendanner, Soltis, & Soltis, 2015). Some polyploid populations are separated from adjacent diploid populations, such as the NJ and ZS tetraploid populations. It is speculated that a primitive genotype was preserved and doubled, and then adapted through the process of polyploidization. Such processes explain the geographic distribution pattern of coexisting polyploidy and diploid populations. Polyploid species have inherent advantages because they can adapt readily to environmental changes and/or occupy new environments. However, whether polyploidization occurred once or multiple times has not yet been determined. Therefore, further work on the origin and evolution of polyploidies in *G. pentaphyllum*, using modern phylogeography based on molecular methods, is necessary. The use of plant sequence fragments to construct geographic genetic distribution patterns for the different genetic backgrounds, and simulations of evolution using statistical population analysis, have the potential to provide further information on the origin and evolutionary history of this species and its polyploidy complexes in the future.

### 4.5 Implications for conservation

Studies of the genetic diversity and genetic structure of species are important components of biodiversity conservation. Preferential conservation of populations with high diversity optimizes the potential of a species to adapt. However, populations with low genetic diversity should be protected from the threats that arise from evolutionary factors. As a traditional medicinal plant in China, *G. pentaphyllum* has a high medicinal value, but it has been listed as a Grade II Key Protected Wild Plant Species by the Chinese government. Cultivation sites, such as Pingli Jiaogulan Base (Ankang, China), have been established to breed *G. pentaphyllum*, but there is still a risk that the wild resource could be depleted. Therefore, both in situ and ex situ measures should be taken to protect *G. pentaphyllum* resources.

Potential measures to protect *G. pentaphyllum* include: (a) education to enhance public awareness and understanding of the importance of wild plants and develop a culture of protection; (b) the establishment of demonstration bases to encourage the public to protect *G. pentaphyllum*; (c) correct usage of *G. pentaphyllum* resources. Natural populations of *G. pentaphyllum* should not be excavated, and its living habitats should be protected; (d) hybridization between cultivated and wild individuals should be prevented to avoid genomic contamination; and (e) in situ measures should be undertaken to protect populations with high levels of genetic diversity (i.e., the polyploidy populations and diploidic HF, WD, and HS populations), and populations that exhibit specific genotypes and private alleles (i.e., ZT, ML, NN, CS, GM, QC2, RJ, GZ, GP, and JY) should be conserved by a combination of in situ and ex situ measures, for example, removal of plants to a park or botanical garden for protection and scientific study. To summarize, the wild populations of *G. pentaphyllum* resources should be protected and developed sustainably to enable continued utilization of this natural resource.

## CONFLICT OF INTEREST

None declared.

## AUTHORS' CONTRIBUTIONS

G.Z. and Z.L. conceived the ideas; X.Z., H.S., J.Y., and L.F. contributed to the sample collection; X.Z. did the experiments, analyzed the data, and written the manuscript. All authors read and approved the final manuscript.

## Data Availability

Details of all species used in this study including sampling information (longitude and latitude) and genetic diversity values are available in Tables A1 and A3. All the microsatellite genotyping data used in this study are available in Dryad.

## APPENDIX 1

**Table A1:** Sampling details of each population of *G. pentaphyllum*

Pop no.	Pop ID	Location	Ploidy level	Sample size (Di‐,Tetra‐, Hexa‐, Octa‐)	Latitude	Longitude
*G. pentaphyllum*						
1	ST	Shitai, Anhui	2×	19 (19,0,0,0)	30°11′N	117°31′E
2	LA	Lu'an, Anhui	2×	24 (24,0,0,0)	31°45′N	116°31′E
3	XC	Xuancheng, Anhui	2×	16 (16,0,0,0)	30°56′N	118°45′E
4	JY	Beibei, Chongqing	2×	16 (16,2,0,0)	29°50′N	106°23′E
5	SX	Shaxian, Fujian	2×	20 (20,0,0,0)	26°24′N	117°47′E
6	GD	Guangzhou, Guangdong	2×	13 (13,0,0,0)	23°10′N	113°16′E
7	GP	Guiping, Guangxi	2×	15 (15,1,0,0)	23°26′N	110°04′E
8	LZ	Liuzhou, Guangxi	2×	13 (13,0,0,0)	24°17′N	109°38′E
9	LP	Liupanshui, Guizhou	2×	17 (17,0,0,0)	26°29′N	104°46′E
10	HN	Wuzhishan, Hainan	2×	12 (12,0,0,0)	18°46′N	109°31′E
11	XY	Xinyang, Henan	2×	19 (19,0,0,0)	32°08′N	114°05′E
12	ES	Enshi, Hubei	2×	15 (15,0,0,0)	30°16′N	109°29′E
13	HF	Hefeng, Hubei	2×	20 (20,8,0,0)	29°53′N	110°01′E
14	FX	Fangxian, Hubei	2×	9 (9,1,0,0)	32°03′N	110°44′E
15	WD	Wudang, Hubei	2×	14 (14,1,0,0)	32°23′N	111°00′E
16	HB	Zhushan, Hubei	2×	11 (11,3,0,0)	32°13′N	110°13′E
17	GZ	Guzhang, Hunan	2×	12 (12,6,0,0)	28°36′N	109°59′E
18	HS	Zhangjiajie, Hunan	2×	9 (9,4,0,0)	29°13′N	110°27′E
19	ZJ	Zhangjiajie, Hunan	2×	14 (14,0,0,0)	29°13′N	110°27′E
20	ZZ	Zhuzhou, Hunan	2×	17 (17,0,0,0)	27°50′N	113°07′E
21	BH	Jurong, Jiangsu	2×	19 (19,1,0,0)	32°07′N	119°04′E
22	DY	Dayu, Jiangxi	2×	16 (16,0,0,0)	25°23′N	114°05′E
23	RJ	Ruijin, Jiangxi	2×	18 (18,0,0,0)	25°51′N	116°03′E
24	WN	Wuning, Jiangxi	2×	18 (18,0,0,0)	29°19′N	115°05′E
25	SR	Shangrao, Jiangxi	2×	15 (15,0,0,0)	28°27′N	117°56′E
26	YX	Pingli, Shaanxi	2×	13 (13,0,0,0)	32°21′N	109°17′E
27	XA	Xi'an, Shaanxi	2×	20 (20,11,0,0)	33°56′N	108°06′E
28	QC	Chengdu, Sichuan	2×	10 (10,0,0,0)	30°55′N	103°34′E
29	QC2	Chengdu, Sichuan	2×	11 (11,0,0,0)	30°55′N	103°34′E
30	EM	Emeishan, Sichuan	2×	10 (10,0,0,0)	29°33′N	103°25′E
31	GA	Guang'an, Sichuan	2×	12 (12,0,0,0)	30°15′N	106°48′E
32	GY	Guangyuan, Sichuan	2×	14 (14,0,0,0)	32°26′N	105°50′E
33	GM	Yanyuan, Sichuan	2×	15 (15,0,0,0)	27°23′N	101°31′E
34	PZ	Panzhihua, Sichuan	2×	13 (13,0,0,0)	26°36′N	101°43′E
35	WY	Wanyuan, Sichuan	2×	20 (20,2,0,0)	31°47′N	107°41′E
36	LJ	Xiachang, Sichuan	2×	15 (15,0,0,0)	27°51′N	102°18′E
37	AL	Jiayi, Taiwan	2×	20 (20,9,0,0)	23°30′N	120°48′E
38	HH	Nantou, Taiwan	2×	20 (20,4,0,0)	23°58′N	120°58′E
39	TC	Tengchong, Yunnan	2×	16 (16,0,0,0)	25°06′N	98°30′E
40	CS	Cangshan, Yunnan	2×	9 (9,0,0,1)	25°50′N	100°10′E
41	CZ	Cizhong, Yunnan	2×	15 (15,0,0,0)	28°01′N	98°54′E
42	JH	Jinghong, Yunnan	2×	11 (11,0,0,0)	21°59′N	100°47′E
43	NN	Jinghong, Yunnan	2×	12 (12,1,0,0)	21°56′N	100°36′E
44	LC	Lincang, Yunnan	2×	18 (18,1,0,0)	23°52′N	100°04′E
45	ML	Mengla, Yunnan	2×	15 (15,0,0,0)	21°33′N	101°34′E
46	TH	Yuxi, Yunnan	2×	11 (11,0,0,0)	24°06′N	102°44′E
47	ZT	Zhaotong, Yunnan	2×	15 (15,0,0,0)	27°21′N	103°43′E
48	WS	Hangzhou, Zhejiang	2×	16 (16,0,0,0)	30°14′N	120°09′E
49	YH	Hangzhou, Zhejiang	2×	14 (14,0,0,0)	30°13′N	120°09′E
50	YN	Ha Giang, Vietnam	2×	9 (9,0,0,0)	22°45′N	104°56′E
51	BS	Baise, Guangxi	4×	15 (0,15,0,0)	23°55′N	106°37′E
52	RH	Renhuai, Guizhou	4×	15 (0,15,0,1)	27°50′N	106°24′E
53	XX	Xixia, Henan	4×	7 (1,7,0,0)	33°17′N	111°28′E
54	XX2	Xixia, Henan	4×	12 (2,12,0,0)	33°17′N	111°28′E
55	WG	Wugang, Henan	4×	15 (1,15,0,0)	33°09′N	113°35′E
56	LH	Linghu, Henan	4×	12 (0,12,0,0)	34°27′N	110°40′E
57	SY	Shiyan, Hubei	4×	12 (0,12,0,0)	32°26′N	110°43′E
58	NJ	Nanjing, Jiangsu	4×	12 (0,12,0,0)	32°06′N	118°48′E
59	ZS	Nanjing, Jiangsu	4×	10 (0,10,0,0)	32°06′N	118°48′E
60	YF	Pingli, Shaanxi	4×	10 (3,10,0,0)	32°21′N	109°17′E
61	YT	Yingtou, Shaanxi	4×	17 (0,17,0,0)	34°09′N	107°45′E
62	WL	Hanzhong, Shaanxi	4×	16 (3,16,0,0)	33°35′N	106°17′E
63	TZ	Shangluo, Shanxi	4×	15 (2,15,0,0)	33°23′N	110°01′E
64	FH	Emeishan, Sichuan	4×	10 (0,10,0,0)	29°33′N	103°25′E
65	LD	Luding, Sichuan	4×	18 (0,18,0,0)	29°57′N	102°13′E
66	KM	Kunming, Yunnan	4×	10 (0,10,0,0)	24°57′N	102°38′E
67	KZ	Kunming, Yunnan	4×	9 (4,9,0,0)	25°09′N	102°44′E
68	SQ	Kunming, Yunnan	4×	10 (0,10,0,0)	24°57′N	102°38′E
69	JS	Jishou, Hunan	6×	17 (0,0,17,0)	28°17′N	109°42′E
70	YJ	Yingjiang, Yunnan	6×	14 (0,0,14,0)	24°36′N	97°39′E
71	DL	Dali, Yunnan	8×	10 (0,0,0,10)	25°38′N	100°16′E
72	DL2	Dali, Yunnan	8×	9 (0,0,0,9)	25°38′N	100°16′E
*Gomphogyne cissiformis*						
73	O1	Yongde, Yunnan	2×	5 (5,0,0,0)	24°11′N	99°30′E
*Gomphogyne cissiformis var. villosa*						
74	O2	Yongde, Yunnan	2×	5 (5,0,0,0)	24°11′N	99°30′E
Total				1,030 (1,103)		

**Table A2:** Results of Hardy–Weinberg equilibrium and linkage disequilibria

Pop No.	Pop ID	Hardy–Weinberg equilibrium										Linkage disequilibria
		SSRE1	SSRE2	SSRE3	SSRE4	SSR1	SSR2	SSR3	SSR4	SSR5	SSR6	
1	NN	0.010	0.880	0.083	0.062	0.970	0.100	0.085	0.080	0.753	M	0.000*
2	HS	0.367	0.860	0.398	0.052	0.038	0.004*	0.722	0.708	0.860	M	0.000*
3	ZJ	0.943	0.038	0.101	0.533	M	0.773	M	0.773	M	0.308	0.000*
4	GD	M	M	M	0.885	0.000*	M	M	0.002*	M	M	0.000*
5	JY	0.007*	0.790	0.003*	0.837	0.459	0.182	0.908	0.568	M	0.356	0.000*
6	WS	0.000*	M	0.001*	0.000*	0.790	0.086	0.459	0.000*	M	M	0.000*
7	ML	0.000*	0.000*	0.000*	0.000*	M	0.239	0.000*	0.000*	M	M	0.000*
8	WN	0.875	0.494	M	0.007	M	0.001*	0.088	M	M	0.007	0.000*
9	DL	0.000*	0.019	0.175	0.002*	0.868	0.429	0.164	0.002*	0.002*	M	0.000*
10	DL2	0.016	0.003*	0.003*	0.003*	0.860	0.708	0.003*	M	0.029	M	0.000*
11	RH	0.000*	0.519	0.000*	M	0.894	0.002*	M	M	M	M	0.000*
12	ZT	0.667	0.258	0.083	0.551	0.894	0.555	0.847	0.085	0.894	0.894	0.000*
13	NJ	0.842	0.753	0.248	0.392	M	0.001*	M	0.665	0.001*	0.248	0.000*
14	ZS	0.868	M	0.002*	M	0.577	0.002*	M	0.429	0.002*	0.175	0.000*
15	QC	0.217	0.024	0.006*	0.774	0.725	0.868	0.868	0.083	M	0.577	0.000*
16	QC2	0.294	0.740	0.749	0.367	0.875	0.036	0.461	0.906	0.875	0.740	0.000*
17	LC	0.338	0.396	0.601	0.157	0.063	0.494	M	0.060	0.494	0.964	0.000*
18	GP	0.424	0.034	0.005*	M	M	0.000*	0.439	0.001*	M	0.424	0.000*
19	JS	0.901	0.000*	0.000*	0.001*	M	0.690	M	0.690	0.690	M	0.000*
20	CZ	M	0.551	0.930	0.001*	M	0.439	0.053	M	M	0.025	0.000*
21	ES	0.894	0.002*	0.782	M	M	0.000*	0.000*	M	0.894	M	0.000*
22	TC	0.897	0.897	0.356	0.129	0.897	0.094	M	0.033	0.790	M	0.000*
23	GY	0.025	M	0.584	0.180	M	0.773	0.360	0.005*	M	M	0.000*
24	YF	0.577	0.725	0.010	0.003*	M	0.103	0.868	0.002*	M	M	0.000*
25	YX	0.000*	M	0.000*	0.000*	0.885	0.000*	0.000*	M	M	M	0.000*
26	BS	0.000*	0.000*	0.002*	0.000*	0.894	0.005*	0.439	M	M	0.000*	0.000*
27	RJ	M	0.000*	M	0.000*	M	0.596	M	M	M	0.803	0.000*
28	XC	M	M	M	M	M	M	M	M	0.000*	M	0.000*
29	YH	0.584	0.000*	0.852	M	M	0.852	0.890	0.000*	M	M	0.000*
30	SQ	M	0.019	0.125	M	M	M	0.002*	0.002*	0.019	M	0.000*
31	KM	M	M	M	M	M	0.002*	0.002*	M	0.002*	M	0.000*
32	HN	M	M	M	M	0.880	M	M	M	0.753	M	0.000*
33	LH	0.002*	0.035	0.003*	0.229	M	0.126	0.154	0.753	M	M	0.000*
34	YN	0.003*	M	M	0.003*	0.134	0.003*	0.003*	M	M	0.003*	0.000*
35	YJ	0.773	0.000*	0.262	0.773	0.773	0.524	0.000*	0.229	0.229	0.533	0.000*
36	YT	0.000*	0.000*	0.000*	0.000*	0.901	0.000*	M	0.000*	0.000*	M	0.000*
37	LP	0.000*	0.000*	0.000*	M	M	0.000*	0.901	0.901	0.996	M	0.000*
38	LJ	0.001*	0.894	M	0.949	0.894	0.053	0.189	0.894	M	0.894	0.000*
39	TH	0.001*	0.001*	M	M	0.875	M	0.001*	M	M	M	0.000*
40	ZZ	M	0.582	0.690	0.002*	M	0.000*	0.049	0.000*	M	M	0.000*
41	SR	M	M	0.000*	M	0.015	0.894	0.000*	M	M	M	0.000*
42	LD	0.000*	0.000*	0.000*	0.000*	0.007	0.000*	M	0.002*	0.000*	M	0.000*
43	CS	0.056	0.003*	0.003*	0.860	0.948	0.000*	0.708	0.003*	0.708	M	0.000*
44	LA	0.484	0.003*	0.001*	0.917	0.917	0.000*	0.656	M	M	0.484	0.000*
45	PZ	0.011	M	M	0.002*	0.638	0.000*	0.000*	0.764	0.885	M	0.000*
46	FH	0.910	0.325	0.000*	0.035	M	0.577	0.098	0.004*	M	0.577	0.000*
47	EM	0.002*	0.002*	0.002*	0.002*	0.868	0.577	0.002*	0.002*	M	M	0.000*
48	SY	0.106	0.001*	0.003*	0.015	0.753	0.182	0.753	0.397	M	0.880	0.000*
49	JH	M	0.001*	0.058	M	M	M	0.001*	0.014	M	M	0.000*
50	XY	0.809	0.120	0.000*	0.809	M	0.032	0.044	0.245	M	M	0.000*
51	FX	0.003*	M	0.000*	M	M	0.000*	0.134	0.708	0.860	M	0.000*
52	XX	0.839	M	0.072	0.008	M	0.008	0.047	0.008	M	M	0.000*
53	XX2	0.125	0.007	0.000*	0.038	M	0.001*	0.621	0.621	M	M	0.000*
54	GM	0.000*	0.000*	M	0.002*	0.439	0.000*	0.239	0.667	M	M	0.000*
55	WY	0.000*	0.717	0.128	0.000*	M	0.717	0.084	0.492	M	0.717	0.000*
56	DY	0.000*	0.000*	0.000*	0.000*	M	0.000*	0.000*	0.000*	M	M	0.000*
57	KZ	0.391	0.708	0.037	0.096	M	0.124	0.141	0.764	0.860	M	0.000*
58	SX	0.000*	0.000*	0.000*	0.000*	0.047	0.000*	0.000*	0.000*	M	M	0.000*
59	AL	0.614	M	0.136	0.061	0.004*	0.036	0.006*	0.069	M	M	0.000*
60	HH	0.909	0.909	0.264	0.048	0.970	M	0.341	0.051	M	0.343	0.000*
61	XA	0.010	M	0.006*	0.136	0.982	0.001*	0.523	0.017	M	M	0.000*
62	HF	0.325	0.037	0.000*	0.005*	0.012	0.000*	0.080	0.053	0.004*	0.103	0.000*
63	LZ	0.279	0.002*	0.005*	0.364	0.999	0.000*	0.000*	0.764	0.140	0.391	0.000*
64	WG	0.056	0.003*	0.000*	0.047	0.667	0.002*	0.002*	0.000*	M	M	0.000*
65	WL	0.347	0.001*	0.041	0.790	0.871	0.006*	0.002*	0.000*	0.790	0.263	0.000*
66	GZ	0.488	0.488	0.000*	0.248	M	0.006*	0.029	0.488	0.753	M	0.000*
67	BH	0.446	0.414	0.135	0.176	0.325	0.120	0.004*	0.414	M	0.709	0.000*
68	WD	0.791	0.001*	0.038	0.003*	M	0.158	0.015	0.024	0.890	0.890	0.000*
69	ST	0.325	M	0.003*	0.039	0.608	0.046	0.453	0.072	M	0.364	0.000*
70	HB	0.601	0.000*	0.006*	0.000*	0.909	0.009	0.000*	0.012	M	M	0.000*
71	TZ	0.086	0.000*	0.032	0.002*	0.921	0.001*	M	0.000*	0.667	0.980	0.000*
72	GA	0.013	0.013	0.013	0.753	M	0.058	0.001*	M	M	0.392	0.000*

Abbreviation: M, monomorphic site.

* Corrected significance levels (*p* < .00693) by the sequential Bonferroni method.

**Table A3:** Summary of *G. pentaphyllum* population genetic parameters

Pop No.	Pop ID	Sample size	NA	Nae	AR	He	Ho	*h*	*I*	PPL (%)	Fi	Bn	Mode‐shift
Diploid		745	2.08	1.60	1.77	0.277	0.315	0.277	0.443	70.40			
1	ST	19	2.50	1.82	2.03	0.337	0.300	0.337	0.570	80.00	0.113	0.125	S
2	LA	24	2.00	1.41	1.62	0.222	0.254	0.222	0.363	80.00	−0.147	0.629	L
3	XC	16	1.10	1.11	1.10	0.052	0.100	0.052	0.069	10.00	−1.000	0.250	S
4	JY^a^	16	2.40	1.57	1.98	0.305	0.302	0.305	0.517	90.00	0.036	0.875	S
5	SX	20	1.80	1.76	1.76	0.377	0.710	0.377	0.518	80.00	−0.926	0.004	S
6	GD	13	1.50	1.14	1.22	0.082	0.115	0.082	0.134	30.00	−0.44	0.875	L
7	GP^a^	15	2.00	1.67	1.83	0.321	0.208	0.321	0.492	70.00	0.364	0.012	S
8	LZ^a^	13	2.80	1.92	2.23	0.432	0.569	0.432	0.697	100.00	−0.335	0.348	L
9	LP	17	1.80	1.45	1.49	0.229	0.424	0.229	0.330	70.00	−0.896	0.289	S
10	HN	12	1.20	1.03	1.09	0.024	0.025	0.024	0.046	20.00	−0.031	1.000	L
11	XY	19	2.10	1.59	1.78	0.282	0.321	0.282	0.452	70.00	−0.144	0.188	L
12	ES	15	1.70	1.38	1.45	0.187	0.327	0.187	0.281	60.00	−0.791	0.422	S
13	HF	20	3.90	2.39	2.74	0.513	0.379	0.513	0.987	100.00	0.301	0.138	S
14	FX	9	1.90	1.52	1.69	0.244	0.287	0.244	0.365	60.00	−0.156	0.281	S
15	WD	14	2.80	1.97	2.19	0.396	0.408	0.396	0.669	90.00	−0.039	0.410	S
16	HB	11	2.70	1.99	2.33	0.391	0.305	0.391	0.689	80.00	0.181	0.422	S
17	GZ^a^	12	2.40	1.90	2.07	0.356	0.294	0.356	0.603	80.00	0.195	0.014	S
18	HS	9	3.10	2.17	2.39	0.397	0.369	0.397	0.692	90.00	0.182	0.752	S
19	ZJ	14	1.90	1.35	1.61	0.214	0.257	0.214	0.344	70.00	−0.212	0.711	L
20	ZZ	17	1.70	1.54	1.61	0.253	0.382	0.253	0.375	60.00	−0.538	0.039	S
21	BH	19	2.30	1.71	2.00	0.357	0.438	0.357	0.575	90.00	−0.194	0.019	S
22	DY	16	2.00	1.83	1.83	0.378	0.669	0.378	0.542	70.00	−0.814	0.004	S
23	RJ^a^	18	1.40	1.25	1.30	0.134	0.233	0.134	0.195	40.00	−0.781	0.156	S
24	WN	18	1.60	1.41	1.52	0.228	0.306	0.228	0.328	60.00	−0.357	0.039	S
25	SR	15	1.40	1.24	1.29	0.129	0.213	0.129	0.186	40.00	−0.697	0.156	S
26	YX	13	1.60	1.55	1.53	0.268	0.508	0.268	0.363	60.00	−0.97	0.016	S
27	XA	20	2.30	1.72	1.90	0.332	0.372	0.332	0.568	70.00	−0.053	0.148	S
28	QC	10	2.50	1.77	2.04	0.321	0.330	0.321	0.541	90.00	−0.029	0.875	L
29	QC2^a^	11	2.70	1.84	2.11	0.371	0.327	0.371	0.611	100.00	0.124	0.615	L
30	FH	10	2.20	1.83	2.04	0.382	0.340	0.382	0.584	80.00	0.116	0.020	S
31	GA	12	2.10	1.68	1.84	0.342	0.475	0.342	0.488	70.00	−0.327	0.020	S
32	GY	14	1.80	1.54	1.65	0.258	0.250	0.258	0.390	60.00	0.032	0.039	S
33	GM^a^	15	1.80	1.60	1.71	0.301	0.500	0.301	0.438	70.00	−0.699	0.012	S
34	PZ	13	2.10	1.71	1.81	0.280	0.246	0.280	0.454	70.00	0.126	0.289	S
35	WY	20	2.20	1.44	1.82	0.257	0.195	0.257	0.437	80.00	0.217	0.809	L
36	LJ	15	1.90	1.36	1.49	0.201	0.193	0.201	0.307	80.00	0.037	0.629	L
37	AL	20	3.00	2.00	2.22	0.371	0.360	0.371	0.667	70.00	0.106	0.188	L
38	HH	20	2.70	1.80	2.01	0.292	0.343	0.292	0.523	80.00	−0.114	0.680	L
39	TC	16	2.00	1.52	1.65	0.234	0.194	0.234	0.378	80.00	0.177	0.680	L
40	CS^a^	9	2.40	1.36	1.97	0.224	0.099	0.224	0.508	90.00	0.386	0.367	S
41	CZ	15	1.60	1.42	1.55	0.239	0.333	0.239	0.341	60.00	−0.417	0.023	S
42	JH	11	1.40	1.27	1.37	0.158	0.082	0.158	0.225	40.00	0.494	0.031	S
43	NN^a^	12	2.70	1.90	2.20	0.390	0.415	0.390	0.640	90.00	0.029	0.590	L
44	LC	18	2.20	1.55	1.86	0.315	0.323	0.315	0.499	90.00	0.004	0.410	S
45	ML^a^	15	2.00	1.40	1.73	0.250	0.113	0.250	0.405	70.00	0.556	0.594	S
46	TH	11	1.40	1.34	1.34	0.166	0.309	0.166	0.226	40.00	−0.943	0.063	S
47	ZT^b^	15	2.20	1.71	1.86	0.359	0.373	0.359	0.527	100.00	−0.041	0.138	L
48	WS	16	1.90	1.32	1.54	0.180	0.087	0.180	0.302	70.00	0.522	0.766	L
49	YH	14	1.60	1.40	1.51	0.224	0.243	0.224	0.319	60.00	−0.089	0.078	S
50	YN	9	1.60	1.64	1.60	0.312	0.567	0.312	0.410	60.00	−0.915	0.008	S
Tetraploid		225	2.15	1.81	1.89	0.339	0.377	0.339	0.510	72.78			
51	BS	15	2.00	1.75	1.81	0.356	0.434	0.356	0.488	80.00	−0.236	0.010	S
52	RH^b^	15	1.90	1.60	1.61	0.219	0.254	0.219	0.285	50.00	−0.16	0.078	S
53	XX	7	1.80	1.71	1.68	0.272	0.314	0.272	0.363	60.00	−0.158	0.055	S
54	XX2	12	2.40	1.93	2.07	0.348	0.360	0.348	0.614	70.00	0.016	0.055	S
55	WG^a^	15	2.40	2.03	2.08	0.419	0.479	0.419	0.652	80.00	−0.142	0.010	S
56	LH	12	2.40	1.92	2.04	0.369	0.412	0.369	0.567	70.00	−0.099	0.020	S
57	SY	12	2.60	2.08	2.15	0.399	0.414	0.399	0.623	90.00	−0.059	0.102	L
58	NJ	12	2.00	1.59	1.79	0.325	0.317	0.325	0.487	80.00	0.026	0.125	S
59	ZS	10	1.70	1.49	1.59	0.266	0.293	0.266	0.381	70.00	−0.114	0.039	S
60	YF	10	2.20	1.70	1.73	0.280	0.282	0.280	0.450	70.00	0.018	0.531	S
61	YT	17	2.10	2.01	1.97	0.399	0.521	0.399	0.498	80.00	−0.323	0.004	S
62	WL	16	2.90	2.02	2.34	0.474	0.447	0.474	0.810	100.00	0.092	0.053	L
63	TZ	15	2.90	2.08	2.26	0.419	0.416	0.419	0.714	90.00	0.03	0.326	L
64	EM	10	1.90	1.75	1.80	0.356	0.447	0.356	0.478	80.00	−0.28	0.010	S
65	LD	18	1.90	1.80	1.83	0.382	0.483	0.382	0.512	80.00	−0.281	0.004	S
66	KM	10	1.40	1.39	1.38	0.167	0.217	0.167	0.208	30.00	−0.333	0.063	S
67	KZ	9	2.30	1.97	2.04	0.361	0.330	0.361	0.583	80.00	0.123	0.156	S
68	SQ	10	1.90	1.82	1.80	0.301	0.363	0.301	0.464	50.00	−0.23	0.016	S
Hexaploid		31	2.20	1.56	1.85	0.284	0.176	0.284	0.476	85.00			
69	JS	17	1.90	1.50	1.67	0.226	0.138	0.226	0.376	70.00	0.337	0.469	L
70	YJ^a^	14	2.50	1.62	2.02	0.342	0.214	0.342	0.576	100.00	0.388	0.461	S
Octaploid		19	2.20	1.99	2.05	0.407	0.395	0.407	0.583	85.00			
71	DL	10	2.40	2.19	2.23	0.455	0.418	0.455	0.660	90.00	0.088	0.007	S
72	DL2	9	2.00	1.79	1.86	0.360	0.372	0.360	0.506	80.00	−0.04	0.014	S
Mean			2.10	1.66	1.81	0.297	0.329	0.297	0.465	71.81			

Note

NA, alleles; Nae, effective alleles; AR (*k* = 8), allelic richness (expected number of alleles among eight gene copies); He, gene diversity corrected for sample size; Ho, observed heterozygosity; Fi, individual inbreeding coefficient; *h*, gene diversity with UNORDERED alleles; *I*, Shannon's Information Index = −1 × Sum (*pi* × Ln (*pi*)) where *pi* is the frequency of the *i*th allele for the population; Bn, bottleneck probability of Wilcoxon one‐tailed test for heterozygote excess under PTM; L, normal L‐shaped distribution; S, shifted mode.

a Populations with one private allele.

b Populations with two private alleles.

## References

[ece35618-bib-0001] Beerli, P. (2005). Comparison of Bayesian and maximum‐likelihood inference of population genetic parameters. Bioinformatics, 22, 341–345. 10.1093/bioinformatics/bti803 16317072

[ece35618-bib-0002] Bohonak, A. (2002). IBD (isolation by distance): A program for analyses of isolation by distance. Journal of Heredity, 93, 153–154. 10.1093/jhered/93.2.153 12140277

[ece35618-bib-0003] Botstein, D. , White, R. L. , Skolnick, M. , & Davis, R. W. (1980). Construction of a genetic linkage map in man using restriction fragment length polymorphisms. American Journal of Human Genetics, 32, 314. 6247908 PMC1686077

[ece35618-bib-0004] Brysting, A. K. , Oxelman, B. , Huber, K. T. , Moulton, V. , & Brochmann, C. (2007). Untangling complex histories of genome mergings in high polyploids. Systematic Biology, 56, 467–476. 10.1080/10635150701424553 17562470

[ece35618-bib-0005] Chen, J. , & Gilbert, M. (2006). Flora of China. St Louis, MO: Beijing and Missouri Botanical Garden Press.

[ece35618-bib-0006] Chen, J.‐M. , Zhao, S.‐Y. , Liao, Y.‐Y. , Gichira, A. W. , Gituru, R. W. , & Wang, Q.‐F. (2015). Chloroplast DNA phylogeographic analysis reveals significant spatial genetic structure of the relictual tree *Davidia involucrata* (Davidiaceae). Conservation Genetics, 16, 583–593. 10.1007/s10592-014-0683-z

[ece35618-bib-0007] Chen, S. (1995). A classificatory system and geographical distribution of the genus Gynostemma BL. (Cucurbitaceae). Acta Phytotaxonomica Sinica, 33, 403–410.

[ece35618-bib-0008] Ciofi, C. , Beaumontf, M. A. , Swingland, I. R. , & Bruford, M. W. (1999). Genetic divergence and units for conservation in the Komodo dragon *Varanus komodoensis* . Proceedings of the Royal Society of London B: Biological Sciences, 266, 2269–2274.

[ece35618-bib-0009] Collins, W. D. , Bitz, C. M. , Blackmon, M. L. , Bonan, G. B. , Bretherton, C. S. , Carton, J. A. , … Smith, R. D. (2006). The community climate system model version 3 (CCSM3). Journal of Climate, 19, 2122–2143. 10.1175/JCLI3761.1

[ece35618-bib-0010] Cui, L. , Wall, P. K. , Leebens‐Mack, J. H. , Lindsay, B. G. , Soltis, D. E. , Doyle, J. J. , … Barakat, A. (2006). Widespread genome duplications throughout the history of flowering plants. Genome Research, 16, 738–749. 10.1101/gr.4825606 16702410 PMC1479859

[ece35618-bib-0011] Di Rienzo, A. , Peterson, A. , Garza, J. , Valdes, A. , Slatkin, M. , & Freimer, N. (1994). Mutational processes of simple‐sequence repeat loci in human populations. Proceedings of the National Academy of Sciences of the United States of America, 91, 3166–3170. 10.1073/pnas.91.8.3166 8159720 PMC43536

[ece35618-bib-0012] Du, F. K. , Hou, M. , Wang, W. , Mao, K. , & Hampe, A. (2017). Phylogeography of *Quercus aquifolioides* provides novel insights into the Neogene history of a major global hotspot of plant diversity in south‐west China. Journal of Biogeography, 44, 294–307.

[ece35618-bib-0013] Earl, D. A. (2012). STRUCTURE Harvester: A website and program for visualizing Structure output and implementing the Evanno method. Conservation Genetics Resources, 4, 359–361. 10.1007/s12686-011-9548-7

[ece35618-bib-0014] Ehrendorfer, F. (1980). Polyploidy and distribution In Lewis W. H. (Ed.), Polyploidy: Biological relevance (pp. 45–60). London: Plenum Press.

[ece35618-bib-0015] Ellstrand, N. C. , & Elam, D. R. (1993). Population genetic consequences of small population size: Implications for plant conservation. Annual Review of Ecology and Systematics, 24, 217–242. 10.1146/annurev.es.24.110193.001245

[ece35618-bib-0016] Evanno, G. , Regnaut, S. , & Goudet, J. (2005). Detecting the number of clusters of individuals using the software STRUCTURE: A simulation study. Molecular Ecology, 14, 2611–2620. 10.1111/j.1365-294X.2005.02553.x 15969739

[ece35618-bib-0017] Excoffier, L. , & Lischer, H. E. (2010). Arlequin suite ver 3.5: A new series of programs to perform population genetics analyses under Linux and Windows. Molecular Ecology Resources, 10, 564–567. 10.1111/j.1755-0998.2010.02847.x 21565059

[ece35618-bib-0018] Feng, L. , Zheng, Q.‐J. , Qian, Z.‐Q. , Yang, J. , Zhang, Y.‐P. , Li, Z.‐H. , & Zhao, G.‐F. (2016). Genetic structure and evolutionary history of three Alpine Sclerophyllous oaks in East Himalaya‐Hengduan Mountains and adjacent regions. Frontiers in Plant Science, 7, 1688 10.3389/fpls.2016.01688 27891142 PMC5104984

[ece35618-bib-0019] Fielding, A. H. , & Bell, J. F. (1997). A review of methods for the assessment of prediction errors in conservation presence/absence models. Environmental Conservation, 24, 38–49. 10.1017/S0376892997000088

[ece35618-bib-0020] Foll, M. (2012). BayeScan v2. 1 user manual. Ecology, 20, 1450–1462.

[ece35618-bib-0021] Gao, X.‐F. , Chen, S.‐K. , Gu, Z. , & Zhao, J.‐Z. (1995). A chromosomal study on the genus *Gynostemma* (Cucurbitaceae). Acta Botanica Yunnanica, 17, 312–316.

[ece35618-bib-0022] Gavin, D. G. , Fitzpatrick, M. C. , Gugger, P. F. , Heath, K. D. , Rodríguez‐Sánchez, F. , Dobrowski, S. Z. , … Williams, J. W. (2014). Climate refugia: Joint inference from fossil records, species distribution models and phylogeography. New Phytologist, 204, 37–54. 10.1111/nph.12929 25039238

[ece35618-bib-0023] Genton, B. , Shykoff, J. , & Giraud, T. (2005). High genetic diversity in French invasive populations of common ragweed, *Ambrosia artemisiifolia*, as a result of multiple sources of introduction. Molecular Ecology, 14, 4275–4285. 10.1111/j.1365-294X.2005.02750.x 16313592

[ece35618-bib-0024] Goudet, J. (2002). FSTAT 2.9. 3: A program to estimate and test gene diversities and fixation indices (updated from Goudet 1995). Lausanne, Switzerland.

[ece35618-bib-0025] Grant, V. (1985). The evolutionary process: A critical study of evolutionary theory. New York, NY: Columbia University Press.

[ece35618-bib-0026] Hardy, O. J. , & Vekemans, X. (2002). SPAGeDi: A versatile computer program to analyse spatial genetic structure at the individual or population levels. Molecular Ecology Notes, 2, 618–620. 10.1046/j.1471-8286.2002.00305.x

[ece35618-bib-0027] Hewitt, G. (2000). The genetic legacy of the Quaternary ice ages. Nature, 405, 907 10.1038/35016000 10879524

[ece35618-bib-0028] Hijmans, R. J. , Cameron, S. E. , Parra, J. L. , Jones, P. G. , & Jarvis, A. (2005). Very high resolution interpolated climate surfaces for global land areas. International Journal of Climatology, 25, 1965–1978. 10.1002/joc.1276

[ece35618-bib-0029] Huang, K. , Ritland, K. , Dunn, D. W. , & Li, B. (2019). Individual ploidy assignment and estimating the allele frequency in polysomic in‐heritance. Submitting.

[ece35618-bib-0030] Hulce, D. , Li, X. , Snyder‐Leiby, T. , & Liu, C. J. (2011). GeneMarker^®^ genotyping software: Tools to Increase the statistical power of DNA fragment analysis. Journal of Biomolecular Techniques, 22, S35.

[ece35618-bib-0031] Jensen, J. L. , Bohonak, A. J. , & Kelley, S. T. (2005). Isolation by distance, web service. BMC Genetics, 6, 13. 15760479 10.1186/1471-2156-6-13 PMC1079815

[ece35618-bib-0032] Jiang, L.‐Y. , Qian, Z.‐Q. , Guo, Z.‐G. , Wang, C. , & Zhao, G.‐F. (2009). Polyploid origins in *Gynostemma pentaphyllum* (Cucurbitaceae) inferred from multiple gene sequences. Molecular Phylogenetics and Evolution, 52, 183–191. 10.1016/j.ympev.2009.03.004 19292995

[ece35618-bib-0033] Knox, J. S. , Bezold, K. , Cabe, P. R. , Williams, S. , & Simurda, M. C. (2016). Genetic diversity and population structure of the endemic disjunct species, *Helenium virginicum* (Asteraceae). The American Midland Naturalist, 175, 242–260. 10.1674/0003-0031-175.2.242

[ece35618-bib-0034] Kohn, M. H. , York, E. C. , Kamradt, D. A. , Haught, G. , Sauvajot, R. M. , & Wayne, R. K. (1999). Estimating population size by genotyping faeces. Proceedings of the Royal Society of London B: Biological Sciences, 266, 657–663. 10.1098/rspb.1999.0686 PMC1689828 10331287

[ece35618-bib-0035] Li, J. , Wan, Q. , Guo, Y. P. , Abbott, R. J. , & Rao, G. Y. (2014). Should I stay or should I go: Biogeographic and evolutionary history of a polyploid complex (*Chrysanthemum indicum* complex) in response to Pleistocene climate change in China. New Phytologist, 201, 1031–1044. 24400906 10.1111/nph.12585

[ece35618-bib-0036] Li, Z.‐H. , Liu, Z.‐L. , Zhao, P. , Su, H.‐L. , & Zhao, G.‐F. (2012). A review on studies of systematic evolution of *Gynostemma* Bl. Acta Botanica Boreali‐Occidentalia Sinica, 32, 2133–2138.

[ece35618-bib-0037] Liao, H. , Zhao, Y. , Zhou, Y. , Wang, Y. , Wang, X. , Lu, F. , & Song, Z. (2011). Microsatellite markers in the traditional Chinese medicinal herb *Gynostemma pentaphyllum* (Cucurbitaceae). American Journal of Botany, 98, e61–e63. 10.3732/ajb.1000456 21613126

[ece35618-bib-0039] Lischer, H. E. , & Excoffier, L. (2011). PGDSpider: An automated data conversion tool for connecting population genetics and genomics programs. Bioinformatics, 28, 298–299. 10.1093/bioinformatics/btr642 22110245

[ece35618-bib-0040] Liu, J. , Möller, M. , Provan, J. , Gao, L. M. , Poudel, R. C. , & Li, D. Z. (2013). Geological and ecological factors drive cryptic speciation of yews in a biodiversity hotspot. New Phytologist, 199, 1093–1108. 10.1111/nph.12336 23718262

[ece35618-bib-0041] Loveless, M. D. , & Hamrick, J. L. (1984). Ecological determinants of genetic structure in plant populations. Annual Review of Ecology and Systematics, 15, 65–95. 10.1146/annurev.es.15.110184.000433

[ece35618-bib-0042] Luikart, G. , Allendorf, F. , Cornuet, J. , & Sherwin, W. (1998). Distortion of allele frequency distributions provides a test for recent population bottlenecks. Journal of Heredity, 89, 238–247. 10.1093/jhered/89.3.238 9656466

[ece35618-bib-0043] Lumaret, R. , Guillerm, J.‐L. , Maillet, J. , & Verlaque, R. (1997). Plant species diversity and polyploidy in islands of natural vegetation isolated in extensive cultivated lands. Biodiversity & Conservation, 6, 591–613.

[ece35618-bib-0044] Maceira, N. O. , Jacquard, P. , & Lumaret, R. (1993). Competition between diploid and derivative autotetraploid *Dactylis glomerata* L. from Galicia. Implications for the establishment of novel polyploid populations. New Phytologist, 124, 321–328. 10.1111/j.1469-8137.1993.tb03822.x 33874356

[ece35618-bib-0045] Mantel, N. (1967). The detection of disease clustering and a generalized regression approach. Cancer Research, 27, 209–220. 6018555

[ece35618-bib-0046] McIntyre, P. J. (2012). Polyploidy associated with altered and broader ecological niches in the *Claytonia perfoliata* (Portulacaceae) species complex. American Journal of Botany, 99, 655–662. 22434773 10.3732/ajb.1100466

[ece35618-bib-0047] Mellick, R. , Lowe, A. , Allen, C. , Hill, R. S. , & Rossetto, M. (2012). Palaeodistribution modelling and genetic evidence highlight differential post‐glacial range shifts of a rain forest conifer distributed across a latitudinal gradient. Journal of Biogeography, 39, 2292–2302. 10.1111/j.1365-2699.2012.02747.x

[ece35618-bib-0048] Morris, A. B. , Ickert‐Bond, S. M. , Brunson, D. B. , Soltis, D. E. , & Soltis, P. S. (2008). Phylogeographical structure and temporal complexity in American sweetgum (*Liquidambar styraciflua*; Altingiaceae). Molecular Ecology, 17, 3889–3900. 18662227 10.1111/j.1365-294X.2008.03875.x

[ece35618-bib-0049] Nei, M. (1972). Genetic distance between populations. The American Naturalist, 106, 283–292. 10.1086/282771

[ece35618-bib-0050] Nielsen, R. (2005). Molecular signatures of natural selection. Annual Review of Genetics, 39, 197–218. 10.1146/annurev.genet.39.073003.112420 16285858

[ece35618-bib-0051] Nolan, C. , Noyes, A. , Bennett, A. , Hunter, R. , & Hunter, K. (2010). Inter simple sequence repeats (ISSR) reveal genetic variation among mid‐Atlantic populations of threatened *Amaranthus pumilus* and phylogenetic relationships. Castanea, 75, 506–516. 10.2179/09-050.1

[ece35618-bib-0052] Ortego, J. , Noguerales, V. , Gugger, P. F. , & Sork, V. L. (2015). Evolutionary and demographic history of the Californian scrub white oak species complex: An integrative approach. Molecular Ecology, 24, 6188–6208. 10.1111/mec.13457 26547661

[ece35618-bib-0053] Otto, S. P. , & Whitton, J. (2000). Polyploid incidence and evolution. Annual Review of Genetics, 34, 401–437. 10.1146/annurev.genet.34.1.401 11092833

[ece35618-bib-0054] Pang, M. , Zou, F.‐P. , & Xiao, Y.‐P. (2006). Construction of DNA fingerprint for *Gynostemma pentaphyllum* (Thunb) Makino based on RAPD analysis. Journal of Shaanxi Normal University (Natural Science Edition), 3, 025.

[ece35618-bib-0055] Parisod, C. , & Besnard, G. (2007). Glacial in situ survival in the Western Alps and polytopic autopolyploidy in *Biscutella laevigata* L. (Brassicaceae). Molecular Ecology, 16, 2755–2767. 10.1111/j.1365-294X.2007.03315.x 17594445

[ece35618-bib-0056] Parisod, C. , Holderegger, R. , & Brochmann, C. (2010). Evolutionary consequences of autopolyploidy. New Phytologist, 186, 5–17. 10.1111/j.1469-8137.2009.03142.x 20070540

[ece35618-bib-0057] Peakall, R. , & Smouse, P. E. (2006). GENALEX 6: Genetic analysis in Excel. Population genetic software for teaching and research. Molecular Ecology Notes, 6, 288–295. 10.1111/j.1471-8286.2005.01155.x PMC3463245 22820204

[ece35618-bib-0058] Pembleton, L. W. , Cogan, N. O. , & Forster, J. W. (2013). St AMPP: An R package for calculation of genetic differentiation and structure of mixed‐ploidy level populations. Molecular Ecology Resources, 13, 946–952. 10.1111/1755-0998.12129 23738873

[ece35618-bib-0059] Phillips, S. J. , Anderson, R. P. , & Schapire, R. E. (2006). Maximum entropy modeling of species geographic distributions. Ecological Modelling, 190, 231–259. 10.1016/j.ecolmodel.2005.03.026

[ece35618-bib-0060] Phillips, S. J. , & Dudík, M. (2008). Modeling of species distributions with Maxent: New extensions and a comprehensive evaluation. Ecography, 31, 161–175. 10.1111/j.0906-7590.2008.5203.x

[ece35618-bib-0061] Piry, S. , Luikart, G. , & Cornuet, J. (1999). BOTTLENECK: A computer program for detecting recent reductions in the effective population size using allele frequency data. Journal of Heredity, 90, 502–503.

[ece35618-bib-0062] Pritchard, J. K. , Stephens, M. , & Donnelly, P. (2000). Inference of population structure using multilocus genotype data. Genetics, 155, 945–959. 10835412 10.1093/genetics/155.2.945 PMC1461096

[ece35618-bib-0063] Qiu, Y.‐X. , Fu, C.‐X. , & Comes, H. P. (2011). Plant molecular phylogeography in China and adjacent regions: Tracing the genetic imprints of Quaternary climate and environmental change in the world's most diverse temperate flora. Molecular Phylogenetics and Evolution, 59, 225–244. 10.1016/j.ympev.2011.01.012 21292014

[ece35618-bib-0064] Ramsey, J. (2011). Polyploidy and ecological adaptation in wild yarrow. Proceedings of the National Academy of Sciences of the United States of America, 108, 7096–7101. 10.1073/pnas.1016631108 21402904 PMC3084070

[ece35618-bib-0065] Ramsey, J. , & Ramsey, T. S. (2014). Ecological studies of polyploidy in the 100 years following its discovery. Philosophical Transactions of the Royal Society of London. Series B, Biological Sciences, 369, 20130352. 24958925 10.1098/rstb.2013.0352 PMC4071525

[ece35618-bib-0066] Razmovski‐Naumovski, V. , Huang, T.‐H.‐W. , Tran, V. H. , Li, G. Q. , Duke, C. C. , & Roufogalis, B. D. (2005). Chemistry and pharmacology of *Gynostemma pentaphyllum* . Phytochemistry Reviews, 4, 197–219. 10.1007/s11101-005-3754-4

[ece35618-bib-0067] Rice, W. R. (1989). Analyzing tables of statistical tests. Evolution, 43, 223–225. 10.1111/j.1558-5646.1989.tb04220.x 28568501

[ece35618-bib-0068] Robledo‐Arnuncio, J. J. , Klein, E. K. , Muller‐Landau, H. C. , & Santamaría, L. (2014). Space, time and complexity in plant dispersal ecology. Movement Ecology, 2, 16 10.1186/s40462-014-0016-3 25709828 PMC4337469

[ece35618-bib-0069] Rosenberg, N. A. (2004). DISTRUCT: A program for the graphical display of population structure. Molecular Ecology Notes, 4, 137–138. 10.1046/j.1471-8286.2003.00566.x

[ece35618-bib-0070] Sang, T. (2002). Utility of low‐copy nuclear gene sequences in plant phylogenetics. Critical Reviews in Biochemistry and Molecular Biology, 37, 121–147. 10.1080/10409230290771474 12139440

[ece35618-bib-0071] Saunders, D. A. , Hobbs, R. J. , & Margules, C. R. (1991). Biological consequences of ecosystem fragmentation: A review. Conservation Biology, 5, 18–32. 10.1111/j.1523-1739.1991.tb00384.x

[ece35618-bib-0072] Schlaepfer, D. R. , Edwards, P. J. , & Billeter, R. (2010). Why only tetraploid *Solidago gigantea* (Asteraceae) became invasive: A common garden comparison of ploidy levels. Oecologia, 163, 661–673. 10.1007/s00442-010-1595-3 20238128

[ece35618-bib-0073] Schlötterer, C. (2000). Evolutionary dynamics of microsatellite DNA. Chromosoma, 109, 365–371. 10.1007/s004120000089 11072791

[ece35618-bib-0074] Scoble, J. , & Lowe, A. J. (2010). A case for incorporating phylogeography and landscape genetics into species distribution modelling approaches to improve climate adaptation and conservation planning. Diversity and Distributions, 16, 343–353. 10.1111/j.1472-4642.2010.00658.x

[ece35618-bib-0075] Selkoe, K. A. , & Toonen, R. J. (2006). Microsatellites for ecologists: A practical guide to using and evaluating microsatellite markers. Ecology Letters, 9, 615–629. 10.1111/j.1461-0248.2006.00889.x 16643306

[ece35618-bib-0076] Servick, S. , Visger, C. J. , Gitzendanner, M. A. , Soltis, P. S. , & Soltis, D. E. (2015). Population genetic variation, geographic structure, and multiple origins of autopolyploidy in *Galax urceolata* . American Journal of Botany, 102, 973–982. 10.3732/ajb.1400554 26101421

[ece35618-bib-0077] Slatkin, M. (1985). Gene flow in natural populations. Annual Review of Ecology and Systematics, 393–430. 10.1146/annurev.es.16.110185.002141

[ece35618-bib-0078] Slatkin, M. (1987). Gene flow and the geographic structure of natural populations. Science, 236, 787–792. 10.1126/science.3576198 3576198

[ece35618-bib-0079] Slatkin, M. (1995). A measure of population subdivision based on microsatellite allele frequencies. Genetics, 139, 457–462. 7705646 10.1093/genetics/139.1.457 PMC1206343

[ece35618-bib-0080] Soltis, D. E. , & Soltis, P. S. (1999). Polyploidy: Recurrent formation and genome evolution. Trends in Ecology & Evolution, 14, 348–352. 10.1016/S0169-5347(99)01638-9 10441308

[ece35618-bib-0081] Soltis, P. S. (2005). Ancient and recent polyploidy in angiosperms. New Phytologist, 166, 5–8. 10.1111/j.1469-8137.2005.01379.x 15760346

[ece35618-bib-0082] Spencer, C. , Neigel, J. , & Leberg, P. (2000). Experimental evaluation of the usefulness of microsatellite DNA for detecting demographic bottlenecks. Molecular Ecology, 9, 1517–1528. 10.1046/j.1365-294x.2000.01031.x 11050547

[ece35618-bib-0083] Sullivan, D. M. (2013). Electromagnetic simulation using the FDTD method. Hoboken, NJ: John Wiley & Sons.

[ece35618-bib-0084] Sun, Y. , Hu, H. , Huang, H. , & Vargas‐Mendoza, C. F. (2014). Chloroplast diversity and population differentiation of *Castanopsis fargesii* (Fagaceae): A dominant tree species in evergreen broad‐leaved forest of subtropical China. Tree Genetics & Genomes, 10, 1531–1539. 10.1007/s11295-014-0776-3

[ece35618-bib-0085] te Beest, M. , Le Roux, J. J. , Richardson, D. M. , Brysting, A. K. , Suda, J. , Kubešová, M. , & Pyšek, P. (2011). The more the better? The role of polyploidy in facilitating plant invasions. Annals of Botany, 109, 19–45. 10.1093/aob/mcr277 22040744 PMC3241594

[ece35618-bib-0086] Templeton, A. R. , Shaw, K. , Routman, E. , & Davis, S. K. (1990). The genetic consequences of habitat fragmentation. Annals of the Missouri Botanical Garden, 13–27. 10.2307/2399621

[ece35618-bib-0087] Tremetsberger, K. , König, C. , Samuel, R. , Pinsker, W. , & Stuessy, T. F. (2002). Infraspecific genetic variation in *Biscutella laevigata* (Brassicaceae): New focus on Irene Manton's hypothesis. Plant Systematics and Evolution, 233, 163–181.

[ece35618-bib-0088] Tsai, Y. , Lin, C. , & Chen, B. (2010). Preparative chromatography of flavonoids and saponins in *Gynostemma pentaphyllum* and their antiproliferation effect on hepatoma cell. Phytomedicine, 18, 2–10. 10.1016/j.phymed.2010.09.004 21036575

[ece35618-bib-0089] Van Oosterhout, C. , Hutchinson, W. F. , Wills, D. P. , & Shipley, P. (2004). MICRO‐CHECKER: Software for identifying and correcting genotyping errors in microsatellite data. Molecular Ecology Resources, 4, 535–538.

[ece35618-bib-0090] Waits, L. , Taberlet, P. , Swenson, J. E. , Sandegren, F. , & Franzén, R. (2000). Nuclear DNA microsatellite analysis of genetic diversity and gene flow in the Scandinavian brown bear (*Ursus arctos*). Molecular Ecology, 9, 421–431. 10.1046/j.1365-294x.2000.00892.x 10736045

[ece35618-bib-0091] Wang, C. , Zhang, H. , Qian, Z.‐Q. , & Zhao, G.‐F. (2008). Genetic differentiation in endangered *Gynostemma pentaphyllum* (Thunb.) Makino based on ISSR polymorphism and its implications for conservation. Biochemical Systematics and Ecology, 36, 699–705. 10.1016/j.bse.2008.07.004

[ece35618-bib-0092] Wang, J. , Gao, P. , Kang, M. , Lowe, A. J. , & Huang, H. (2009). Refugia within refugia: The case study of a canopy tree (*Eurycorymbus cavaleriei*) in subtropical China. Journal of Biogeography, 36, 2156–2164.

[ece35618-bib-0093] Wang, Y. H. , Jiang, W. M. , Comes, H. P. , Hu, F. S. , Qiu, Y. X. , & Fu, C. X. (2015). Molecular phylogeography and ecological niche modelling of a widespread herbaceous climber, *Tetrastigma hemsleyanum* (Vitaceae): Insights into Plio‐Pleistocene range dynamics of evergreen forest in subtropical China. New Phytologist, 206, 852–867. 25639152 10.1111/nph.13261

[ece35618-bib-0094] Wilson, G. A. , & Rannala, B. (2003). Bayesian inference of recent migration rates using multilocus genotypes. Genetics, 163, 1177–1191. 12663554 10.1093/genetics/163.3.1177 PMC1462502

[ece35618-bib-0095] Wu, L. L. , Cui, X. K. , Milne, R. I. , Sun, Y. S. , & Liu, J. Q. (2010). Multiple autopolyploidizations and range expansion of *Allium przewalskianum* Regel. (Alliaceae) in the Qinghai‐Tibetan Plateau. Molecular Ecology, 19, 1691–1704. 20345685 10.1111/j.1365-294X.2010.04613.x

[ece35618-bib-0096] Xie, Z. , Liu, W. , Huang, H. , Slavin, M. , Zhao, Y. , Whent, M. , … Yu, L. (2010). Chemical composition of five commercial *Gynostemma pentaphyllum* samples and their radical scavenging, antiproliferative, and anti‐inflammatory properties. Journal of Agricultural and Food Chemistry, 58, 11243–11249. 10.1021/jf1026372 20939605

[ece35618-bib-0097] Yin, F. , Hu, L. , & Pan, R. (2004). Novel dammarane‐type glycosides from *Gynostemma pentaphyllum* . Chemical and Pharmaceutical Bulletin, 52, 1440–1444. 10.1248/cpb.52.1440 15577241

[ece35618-bib-0098] Young, A. , Boyle, T. , & Brown, T. (1996). The population genetic consequences of habitat fragmentation for plants. Trends in Ecology & Evolution, 11, 413–418. 10.1016/0169-5347(96)10045-8 21237900

[ece35618-bib-0099] Yu, Y. (1999). A milestone of wild plant conservation in China. Plants, 5, 3–11.

[ece35618-bib-0100] Zhang, J. , Li, Z. , Fritsch, P. W. , Tian, H. , Yang, A. , & Yao, X. (2015). Phylogeography and genetic structure of a Tertiary relict tree species, *Tapiscia sinensis* (Tapisciaceae): Implications for conservation. Annals of Botany, 116, 727–737. 26187222 10.1093/aob/mcv112 PMC4590324

[ece35618-bib-0102] Zhao, Y.‐M. , Zhou, T. , Li, Z.‐H. , & Zhao, G.‐F. (2015). Characterization of global transcriptome using illumina paired‐end sequencing and development of EST‐SSR markers in two species of *Gynostemma* (Cucurbitaceae). Molecules, 20, 21214–21231. 10.3390/molecules201219758 26633323 PMC6332360

